# Production of 10-methyl branched fatty acids in yeast

**DOI:** 10.1186/s13068-020-01863-0

**Published:** 2021-01-07

**Authors:** Hannah G. Blitzblau, Andrew L. Consiglio, Paulo Teixeira, Donald V. Crabtree, Shuyan Chen, Oliver Konzock, Gamuchirai Chifamba, Austin Su, Annapurna Kamineni, Kyle MacEwen, Maureen Hamilton, Vasiliki Tsakraklides, Jens Nielsen, Verena Siewers, A. Joe Shaw

**Affiliations:** 1 Novogy, Inc., 85 Bolton Street, Cambridge, MA 02140 USA; 2 Present Address: Ginkgo BioWorks, 27 Drydock Ave., Boston, MA 02210 USA; 3 Department of Biology and Biological Engineering, Chalmers University of Technology, Kemivägen 10, 41296 Gothenburg, Sweden; 4 Novo Nordisk Foundation Center for Biosustainability, Chalmers University of Technology, Kemivägen 10, 41296 Gothenburg, Sweden; 5 BioInnovation Institute, Ole Maaløes Vej 3, 2200 Copenhagen N, Denmark; 6 Present Address: Manus Biosynthesis, 1030 Massachusetts Ave. #300, Cambridge, MA 02138 USA

**Keywords:** *Yarrowia lipolytica*, 10-Methylstearic acid, Tuberculostearic acid, Biobased lubricant

## Abstract

**Background:**

Despite the environmental value of biobased lubricants, they account for less than 2% of global lubricant use due to poor thermo-oxidative stability arising from the presence of unsaturated double bonds. Methyl branched fatty acids (BFAs), particularly those with branching near the acyl-chain mid-point, are a high-performance alternative to existing vegetable oils because of their low melting temperature and full saturation.

**Results:**

We cloned and characterized two pathways to produce 10-methyl BFAs isolated from actinomycetes and γ-proteobacteria. In the two-step *bfa* pathway of actinomycetes, BfaB methylates Δ9 unsaturated fatty acids to form 10-methylene BFAs, and subsequently, BfaA reduces the double bond to produce a fully saturated 10-methyl branched fatty acid. A BfaA-B fusion enzyme increased the conversion efficiency of 10-methyl BFAs. The ten-methyl palmitate production (tmp) pathway of γ-proteobacteria produces a 10-methylene intermediate, but the TmpA putative reductase was not active in *E. coli* or yeast. Comparison of BfaB and TmpB activities revealed a range of substrate specificities from C14-C20 fatty acids unsaturated at the Δ9, Δ10 or Δ11 position. We demonstrated efficient production of 10-methylene and 10-methyl BFAs in *S. cerevisiae* by secretion of free fatty acids and in *Y. lipolytica* as triacylglycerides, which accumulated to levels more than 35% of total cellular fatty acids.

**Conclusions:**

We report here the characterization of a set of enzymes that can produce position-specific methylene and methyl branched fatty acids. Yeast expression of *bfa* enzymes can provide a platform for the large-scale production of branched fatty acids suitable for industrial and consumer applications.

## Background

Biobased lubricants account for only a small percentage of the total global lubricant supply [1, 2] despite environmental and sustainability benefits for their use. A major barrier to increased biobased lubricant utilization is that they lack the proper combination of viscosity, low pour point and high oxidation resistance to match petroleum-based and synthetic oils. Most natural fatty acids achieve lower melting temperatures through desaturation, but this leaves them prone to oxidative degradation. Linear saturated long-chain fatty acids with appropriate viscosities have high melting temperatures unsuitable for most lubricant applications. To date, the biobased fatty acid most closely matching the chemical properties of an ideal lubricant is a product termed “isostearic” acid, which is a mixture of branched fatty acids (BFAs) methylated at various positions on the stearate chain [3, 4]. This non-specificity of branch point leads to mixed chemical properties. Isostearic acid is produced by chemical transformation of unsaturated fatty acids, a process that also yields significant amounts of polymer byproducts [4], limiting the production volume and profitability of isostearic acid.

A group of naturally occurring fatty acids achieves lower melting temperatures while maintaining oxidative stability via carbon chain branching on saturated fatty acids. For example, many bacteria including *Escherichia coli* produce cyclopropane fatty acids (CFAs), which have a low melting temperature [5], but the cyclopropane ring is unstable. Iso and anteiso methyl branched fatty acids are produced by *Bacillus subtilis* [6], but the methyl branches located close to the end of the carbon chain do not substantially lower the melting temperature [7]. Fatty acids with random branched sites suitable for biodiesel have been produced from branched-CoA intermediates in *E. coli* and *P. pastoris* by combining the expression of genes from fatty acid and valine and isoleucine biosynthetic pathways [8], however, through this strategy the branch position cannot be controlled. A good candidate for a well-defined, biobased lubricant is 10-methyl stearate. This fully saturated, long-chain fatty acid achieves a low melting temperature (13.2 °C) due to the methyl branch that occurs close to the middle of the fatty acid chain [9]. 10-Methyl fatty acids occur naturally in small amounts in the membranes of a few bacterial species. 10-Methyl stearate, or tuberculostearic acid, was first observed in mycobacterium, and later confirmed to exist in a variety of actinobacteria [10, 11]. 10-Methyl palmitate has also been detected in a subset of γ-proteobacteria [12–14]. Large-scale production of 10-methyl BFAs has been hampered by the fact that these organisms do not produce large amounts of BFAs and are not amenable to commercial production.

The two-step enzymatic production of 10-methylstearic acid was defined through detailed biochemical analysis of *Mycobacterium phlei* [15]. First, a methylase transfers a methyl group from *S*-adenosylmethione (SAM) to the C10 of a phospholipid-bound oleic acid molecule [16]. The reaction is similar to that catalyzed by cyclopropane fatty acid synthase (cfa) enzymes, except the product is a C10-methylene rather than a C9,10 cyclopropane. In a second step, unique to the *bfa* pathway, a reductase saturates the methylene double bond, resulting in the production of 10-methylstearic acid. Although the chemical mechanism of this reaction was proposed 50 years ago [15], the genes responsible for BFA production had not been cloned. Two reports claimed to have identified genes responsible for the production of 10-methylstearic acid [17, 18], but neither gene contained any redox activity to carry out the second step in the production pathway. A recent report identified a two-gene pathway in mycobacteria for the production of 10-methylstearic acid [19] and these genes were functional in cyanobacteria [20].

For large-scale production of branched fatty acids, the oleaginous yeast *Yarrowia lipolytica* and the industrial workhorse *Saccharomyces cerevisiae* are promising host organisms. *Y. lipolytica* naturally accumulates fatty acids in triacyglycerols (TAGs) and has been previously engineered to reach very high lipid contents in bioreactors [21, 22]. Methyl branched fatty acids are produced from an oleic acid precursor, which is highly abundant in engineered *Y. lipolytica* strains [23]. *S. cerevisiae* is a well-known industrial yeast with an extensive literature base and genetic toolkit for advanced engineering. Both *Y. lipolytica* and *S. cerevisiae* have been engineered for enhanced free fatty acid production and secretion [24–26], which could aid the recovery of BFAs.

To enable the production of 10-methyl BFAs, we identified and characterized enzymes that produce site-specific BFAs. Although the major products in yeast are 10-methyl C16 and C18 BFAs, the enzymes methylated substrates with a range chain-lengths and unsaturation sites, expanding the potential product range. Using the *bfa* genes from *Thermomonospora curvata*, we found that fusion of BfaA and BfaB into a single protein increased the production of 10-methyl BFAs. We demonstrated secretion of BFAs in *S. cerevisiae* and accumulation of high levels of BFAs in *Y. lipolytica*.

## Methods

### Gene identification

Bfa and Tmp gene identification began with a review of biochemical literature describing bacterial species with 10-methyl branched fatty acids. A list of representative strains was generated whose genomes were sequenced (Additional file 1: Tables S1 and S2), and the genomes were queried for genes sharing homology to the *E. coli* cyclopropane fatty acid synthase via the GenBank and Metacyc [27] databases. Gene candidates were selected based on their restricted presence in 10-methyl fatty acid producing species and adjacency to a likely redox-active gene. A list of protein sequences and alignments is available in Additional file 3. Candidate gene operons were then cloned in *E. coli* and whole cell biomass assayed for the presence of non-native branched fatty acids.

### *Escherichia coli* expression vector construction

Bacterial genomic DNA was obtained from Deutsche Sammlung von Mikroorganismen und Zellkulturen (DSMZ), Germany. Plasmids were constructed with standard molecular biology techniques using the “yeast gap repair” method [28]. For *E. coli* expression vectors, the empty expression vector pNC53 was restriction digested with enzyme PmeI (New England Biolabs, MA), creating a double strand break between the *tac* promoter and *trpT*’ terminator sequences on this vector. *bfa* gene operons were PCR amplified from genomic DNA with primer flanking sequence such that the *bfaB* ATG start site integrated into the end of the *tac* promoter via homologous recombination. The stop codon of the *bfaA* gene similarly integrated into the beginning of the *trpT*’ terminator region. *E. coli* translation of the operon-embedded *bfaA* gene relied on native translation signals from the donor organism DNA. Where necessary, the first codon of *bfaB* was altered from GTG or TTG to ATG; otherwise the native codon sequence was kept in the *E. coli* expression vectors. Vectors were checked by DNA sequencing and restriction digest for correct construction. All plasmids used in this study are listed in Additional file 1: Table S4.

### Exogenous fatty acid supplementation in *E. coli*

Unsaturated fatty acids were purchased from Nu-Check Prep, Inc., Elysian MN. Fatty acids were dissolved in DMSO at a concentration of 100 mg/mL, with the exceptions of palmitoleic acid, oleic acid, and vaccenic acid, which were dissolved in ethanol at the same concentration. A 10-methylstearic acid reference standard was obtained from Larodan AB, Sweden. Preliminary tests were performed with *E. coli* Top10 (Invitrogen) for evaluation of *bfa* operon expression vectors. Initial screening for active *bfa* and *tmp* operons was performed in 50 mL LB medium supplemented with 100 mg/L ampicillin and 100 mg/L oleic acid at 37 °C and 200 rpm in baffled shake flasks for 41 h. For evaluating conversion of other unsaturated fatty acids, *E. coli* strains NS1161 (empty vector), NS1162 (*T. curvata bfaAB*), NS1237 (*M. hydrocarbonoclasticus tmpBA*), and NS1238 (*T. halophila tmpBA*) were used. NS1161 was constructed by transforming the control plasmid pNC53 into *E. coli* CGSC 9407 (aka JW1653-1 Keio collection) which has a kanR disruption of the native *E. coli* cyclopropane fatty acid synthase (*cfa*) gene. Strains NS1162, NS1237, and NS1238 were constructed in a similar manner by transforming plasmids pNC906, pNC1074, and pNC1076 containing the *bfaAB* operon from *T. curvata* and *tmpBA* operons from *M. hydrocarbonoclasticus*, and *T. halophila*, respectively. *E. coli* strains were grown in LB media supplemented with 100 mg/L ampicillin and 100 mg/L of unsaturated fatty acid with a 5-mL working volume at 37 °C in a rotary drum roller for 24 h. All strains used in this study are described in Additional file 1: Table S5.

### Fatty acid compositional analysis

*Escherichia coli* and yeast cells were harvested by centrifugation, targeting 2–20 mg of lipid in the cell pellet (Tables 1, 2, Additional file 1: Table S3, Additional file 2: Figure S1B, Fig. 5) or the entire volume from the 96-well plate assay (Figs. 2, 3 and 5). Cells were washed twice with 1 mL of deionized water, resuspended in 100 μL deionized water, and frozen at − 80 °C. For Tables 1, 2 and Fig. 2a, cells were lyophilized to dryness and subjected to acid-catalyzed transesterification using 1.25 M hydrochloric acid in methanol (Sigma) at 85 °C for 90 min with mixing by vortexing at 30 min and 60 min to produce fatty acid methyl esters (FAMEs). During the course of this study, we identified acid-catalyzed degradation products arising from 10-methylene fatty acids, similar to that reported earlier for cyclopropane fatty acids [29, 30]. We later determined that base (sodium methoxide) catalyzed transesterification did not cause degradation, and this method was used for all other analyses. In the base-catalyzed transesterification 250 μL of 0.5 M sodium methoxide in methanol (Acros Organics) was added to the cell pellets and incubated at 50 °C for 30 min with mixing by vortexing at 15 min. 250 μL of 0.6 N hydrochloric acid in water and 1 mL isooctane were then added to each sample and mixed by pipetting. The tubes or plate were centrifuged at 2500×*g* at 22 °C to separate the organic and aqueous layers. A sample of the FAME-containing isooctane layer (top layer) was analyzed by gas chromatography equipped with a flame ionization detector (GC-FID) (Agilent Technologies 7890B GC) and VF-23 ms capillary column (20 m × 0.15 mm × 0.15 µm, Agilent Technologies). The FAME-containing sample was injected in the liner at 335 °C with a split ratio of 20:1. The column flow was constant at 0.35 mL/min He. The initial oven temperature was 120 °C, which was then raised to 130 °C at 5 °C/min, and then to 220 °C at 6 °C/min, finally to 240 °C at 40 °C/min and held for 1.5 min. The compounds of interest were identified by comparison of retention times with authentic standards.

**Table 1:** BFA production in *bfaAB* and *tmpBA* expressing *E. coli*

*E. coli* vector	Donor organism	% oleic acid conversion to 10-methylstearic acid
	*bfaAB* operon	
pNC704	*Mycobacterium smegmatis*	4.9 ± 0.6%
pNC721	*Mycobacterium vanbaaleni*	0
pNC755	*Amycolicicoccus subflavus*	0
pNC757	*Corynebacterium glyciniphilum*	0
pNC904	*Rhodococcus opacus*	1.2 ± 0.2%
pNC905	*Thermobifida fusca*	22.0 ± 0.3%
pNC906	*Thermomonospora curvata*	38.3 ± 0.5%
pNC907	*Corynebacterium glutamicum*	0
pNC908	*Agromyces subbeticus*	0
pNC910	*Mycobacterium gilvum*	0
pNC911	*Mycobacterium* sp. *indicus*	0
pNC53	Empty control vector	0

	*tmpBA* operon	% palmitoleic acid conversion to 10-methylenepalmitic acid
pNC1071	*Desulfobacter postgatei*	31.5 ± 0.1%
pNC1072	*Desulfobacter balticum*	0
pNC1073	*Desulfobacula toluolica*	11.8 ± 0.3%
pNC1074	*Marinobacter hydrocarbonoclasticus*	45.3 ± 0.3%
pNC1076	*Thiohalospira halophila*	70.4 ± 0.3%
pNC53	Empty control vector	0

*E. coli* Top10 cells containing the indicated plasmid were cultivated in LB medium supplemented with 100 mg/L oleic acid (for *bfaAB* operons) or 100 mg/L palmitoleic acid (for *tmpBA* operons) and antibiotic. Percent conversion was measured by dividing the 10-methyl/10-methylene fatty acid content by the sum of the remaining precursor fatty acid and the 10-methyl/10-methylene fatty acids present in *E. coli* cell mass

**Table 2:** Acyl-chain substrate percent conversion of heterologous *T. curvata bfa*, *M. hydrocarbonoclasticus tmp*,* and T. halophila tmp* gene operons expressed in *E. coli*

Exogenous fatty acid	*T. curvata bfaAB*	*M. hydrocarbonoclasticus tmpBA*	*T. halophila tmpBA*
12:1Δ11	–	–	–
13:1Δ12	–	–	–
14:1Δ9	3.4%	89%	95%
15:1Δ10	1.7%	86%	69%
16:1Δ9	30.4%	55%	95%
17:1Δ10	11.1%	36%	19%
18:1Δ6	–	–	–
18:1Δ9	33.7%	42%	47%
18:1Δ11	21.8%	9%	8%
18:1Δ9, 12-OH	–	–	–
18:1Δ9, 12	–	–	–
19:1Δ7	–	–	–
19:1Δ10	6.1%	–	–
20:1Δ5	–	–	–
20:1Δ8	–	–	–
20:1Δ11	2.2%	–	–
22:1Δ13	–	–	–
24:1Δ15	–	–	–

Exogenous fatty acids were separately fed to Δ*cfa E. coli* expressing the *T. curvata bfa*, *M. hydrocarbonoclasticus tmp*, and *T. halophila tmp* gene operons and a reference strain with a control vector. FAME profiles were compared across strains and to a control medium with no fatty acid supplementation. Conversion of the exogenously fed fatty acid was calculated as the combined percentage of 10-methyl and 10-methylene fatty acid peak areas relative to the sum of the unsaturated and branched fatty acid peak areas. –: not detected

### Methylene fatty acid identification

*Yarrowia lipolytica* strain NS1165, expressing *T. curvata bfaB* under control of the *Y. lipolytica* TEF1 promoter, and the parental strain NS1009 were cultured in bioreactors before harvesting, washing, freezing, and lyophilization to dryness followed by lipid extraction by bead milling in chloroform–methanol. Extracted material was analyzed by ^13^C nuclear magnetic resonance (NMR), and by GC-FID after sodium methoxide catalyzed transesterification.

### BfaA co-factor assay

*Escherichia coli* strains NS1161, NS1163, and NS1164 were used in this experiment. Strain NS1163 was constructed by transforming plasmid pNC963, containing the *T. curvata bfaB* gene under control of the constitutive *tac* promoter, into *E. coli* CGSC 9407. Strain NS1164 was constructed by transforming plasmid pNC964, containing the *T. curvata bfaA* gene under control of the constitutive *tac* promoter, into *E. coli* CGSC 9407.

Strain NS1163 was grown in 2 × 500 mL LB media in 2 L baffled flasks supplemented with 100 mg/L ampicillin for 24 h at 37 °C. After cultivation, cells were harvested by centrifugation at 3000×*g* for 15 min in an Eppendorf 5810 R clinical centrifuge and washed twice in 100 mL PBS buffer. After concentration to 40 mL PBS buffer, cells were heat inactivated at 85 °C for 30 min. Inactivated cells were then dispensed into 1 mL aliquots and disrupted with 0.3 g of 0.1 mm glass beads using a MP fastprep-24 on “*E. coli*” setting (MP biomedicals, LLC). Whole cell lysed suspension was collected by micro-centrifugation at 2000×*g* for 30 s to remove beads and then 0.7 mL of suspension per tube was transferred to new tubes and frozen at − 80 °C until further use.

On the day of assay, strains NS1161 and NS1164 were grown via inoculation from overnight cultures (1:1000 dilution) in 50 mL LB medium supplemented with 100 mg/L ampicillin in 37 °C and 200 rpm in baffled shake flasks. After 4 h of cultivation, cells were harvested at 5 °C, washed 1× in ice cold PBS and then resuspended in 750 μL PBS in 1 mL plastic screw tubes. 0.3 g of 0.1 mm glass beads were added, and cells were lysed with a MP fastprep-24 on the “*E. coli*” setting. The cell suspension was then micro-centrifuged for 5 min at 12,000×*g*, and the supernatant transferred to a fresh tube and held on ice until assay. The 1 mL reaction contained 700 μL of NS1163 whole lysate with 10 mM NADPH, 10 mM NADH solution, and 100 μL of cell free extract as dictated by the assay conditions. Omitted components were replaced by PBS buffer. Assay tubes were sealed and rotated on a drum roller at 37 °C for 16 h. To end the assay, tubes were frozen at − 80 °C, then lyophilized to dryness followed by in situ extraction and transesterification with methanolic HCl. Fatty acid profiles were determined by GC with flame ionization detection, and the 10-methyl fatty acid peak area was compared to the total fatty acid peak area to determine assay activity.

### *Yarrowia lipolytica* strains and cultivation

All *Y. lipolytica* strains are modifications of the wild-type strain YB-392 from the ARS collection (https://nrrl.ncaur.usda.gov) and are listed in Additional file 1: Table S5. Gene deletions (*tgl3* and *fad2*) were carried out similar to our previous work [31]. Targeting to the designated locus was increased by treating the cells with hydroxyurea to synchronize the cells in S phase, using the local promoter (TGL3 or FAD2) to drive the NAT marker, which reduces the selection frequency of random integration events, and adding 2 kb of homology downstream of the targeted gene. To allow for marker removal, this cassette also contained the negatively selectable hsvTDK gene, followed by a 450-bp direct repeat of homology sequences from the target gene promoter that are upstream of the sequence used for integrations, which enables the full removal of all non-native DNA from the deletion locus. Integration of the disruption construct was selected with 500 mg/L nourseothricin on YPD plates, and gene deletions were verified by PCR. Selective marker removal was carried out by growth on YPD plates (10 g/L yeast extract, 20 g/L Bacto Peptone, 20 g/L glucose, and 20 g/L agar), followed by selection on YPD with 30 μM 5-fluoro, 2-deoxyuridine (FUDR). Marker removal was verified by failure to grow on YPD with nourseothrycin and PCR of the locus. Gene overexpression of the native sequences of all transgenes was carried out by random integration of an expression construct containing the gene of interest and either the HYG or NAT gene and the hsvTDK gene. The positive and negative marker genes were flanked by identical 406 bp sequences of the *Y. lipolytica* *TEF1* promoter, which allowed for recombination-driven marker removal. These *TEF1* promoter regions drove expression of the positive marker and overexpression gene of interest, and successful recombination left a single *TEF1* promoter driving the gene of interest on the chromosome. Marker removal was verified by failure to grow on the positive antibiotic marker and overexpression phenotypes were confirmed after marker removal to ensure the transgene remained in the genome. All plasmids from this study are listed in Additional file 1: Table S4.

*Yarrowia lipolytica* transformations were carried out by a standard lithium acetate heat shock transformation [22]. Cells were pre-grown on solid or liquid YPD overnight. For gene deletions, cells were further incubated in liquid YPD containing 50 mM hydroxyurea for 4 h at 30 °C. For transformation, cells were incubated with transforming DNA, 80 μL 60% polyethylene glycol 4000, 5 μL 2 M dithiothreitol, 5 μL 2 M lithium acetate pH 6, and 2 μL 10 mg/mL single-stranded salmon sperm DNA for 1 h at 39 °C. Cells were outgrown in YPD overnight before plating on selective media (300 mg/L hygromycin or 500 mg/L nourseothricin).

### BFA plate growth and fatty acid analysis

Individual transformant colonies were inoculated into 2-mL deep-well plates containing 300 μL per well of shake-flask media (0.5 g/L urea, 1.5 g/L yeast extract, 0.85 g/L casamino acids, 1.7 g/L yeast nitrogen base without amino acids and ammonium sulfate, 100 g/L glucose and 5.1 g/L potassium hydrogen phthalate to adjust the pH to 5.5). Cells were grown for 96 h at 30 °C at 900 rpm at 70–90% humidity. Cell pellets were washed once with water, freeze-dried and whole cells were subjected to transesterification and fatty acids were analyzed by gas chromatography (GC-FID).

### *Saccharomyces cerevisiae* plasmid and strain construction

Strain CEN.PK113-5D strain was obtained from EUROSCARF (Frankfurt, Germany), strain MLM1.0 was obtained from the authors of Ferreira et al. [32], and strain Y&Z001 was obtained from the authors of Zhou et al. [24]. The *bfaA-B* gene codon-optimized for *S. cerevisiae* (see Additional file 3 Sequences and alignments) was cloned into p416 and p426 [33] using the Gibson Assembly® Master Mix (New England Biolabs) with a synthetic gene fragment (GenScript, NJ, US) containing overlapping sequences to the plasmids linearized by digestion with BamHI and XhoI. *S. cerevisiae* strains were transformed using a PEG/LiAc technique [34].

### Growth medium

*Saccharomyces cerevisiae* strains with auxotrophies were grown on YPD plates. *URA3* plasmid carrying strains were grown on selective growth medium containing 6.9 g/L yeast nitrogen base without amino acids (Formedium, Hunstanton, UK), 0.77 g/L complete supplement mixture without uracil (Formedium), 20 g/L glucose and 20 g/L agar. Shake flask cultivations were performed in minimal medium containing 20 g/L glucose, 5 g/L (NH_4_)_2_SO_4_, 14.4 g/L KH_2_PO_4_, 0.5 g/L MgSO_4_∙7H_2_O adjusted to pH 6. After sterilization, 2 mL/L trace element solution [35] and 1 mL/L of vitamin solution were added.

### *Saccharomyces cerevisiae* shake-flask cultivations

Biological triplicates were pre-cultivated in 5 mL minimal medium at 200 rpm and 30 °C for 18 h. Subsequently, the pre-culture was diluted into 15 mL minimal medium in a 100-mL shake flask to an OD_600_ of 0.1. Shake flasks were incubated at 200 rpm and 30 °C for 72 h. A spectrophotometer (Genesis 20, Thermo Fisher Scientific, Waltham, MA, USA) was used to measure cell density at the end of the shake-flask cultivations.

### Quantification of free fatty acids

Free fatty acids (FFA) were simultaneously extracted and methylated by dichloromethane containing methyl iodide as methyl donor [36]. Briefly, 200 μL aliquots of whole cell culture were taken into glass vials, then 10 μL 40% tetrabutylammonium hydroxide (base catalyst) was added immediately followed by addition of 200 μL dichloromethane containing 200 mM methyl iodide as methyl donor and 100 mg/L pentadecanoic acid as an internal standard. The mixtures were shaken for 30 min at 1400 rpm with a vortex mixer, and then centrifuged at 5000×*g* to promote phase separation. A 160 μL dichloromethane layer was transferred into a GC vial with glass insert and evaporated for 4 h to dryness. The extracted methyl esters were resuspended in 160 μL hexane and then analyzed by gas chromatography (Focus GC, ThermoFisher Scientific) equipped with a Zebron ZB-5MS GUARDIAN capillary column (30 m × 0.25 mm × 0.25 μm, Phenomenex) with a flame ionization detector (ThermoFisher Scientific). The GC program was as follows: 50 °C hold for 2 min; ramp to 140 °C at a rate of 30 °C per minute, then ramp to 280 °C at a rate of 10 °C per min, and hold for 3 min. The inlet temperature was kept at 280 °C. The injection volume was 1 μL. The flow rate of the helium carrier gas was set to 1.0 mL/min. Final quantification was performed using Xcalibur software.

### SAM requirement calculations

The minimum estimated requirements for SAM usage during BFA production were calculated assuming only two cellular pathways would use this metabolite: phosphatidylethanolamine (PE) methylation to phosphatidylcholine (PC) and the BFA pathway. This assumption was based on the study of Ye et al*.* [37], demonstrating that knockout of PE methylation reaction led to up to 30-fold accumulation of SAM in the cell. SAM requirement was calculated then according to the following formula:

*n* SAM = *n* PC * 3 + *n* BFA, in which *n SAM* represents moles of SAM required per g of cell biomass, *n PC* is the calculated moles of PC per g of cell biomass calculated from the molecular weight of 1-stearoyl-2-linoleoyl-sn-glycero-3-phosphatidylcholine and the lipid quantification analysis from Ferreira et al. [25] and Zhou et al. [24], and *n BFA* represents the moles of 10-methyl BFA produced per g of cell biomass.

### BfaA-B localization

Cells were grown for 48 h in minimal medium with 2% glucose and then photographed for green fluorescent protein (GFP) fluorescence using standard methods. Nile Red staining was performed as reported by Ciamponi et al. [38].

### 1-L *Y. lipolytica* batch fermentations

Frozen stocks of strains NS1009, NS1227, and NS1647 were patched onto each of two YPD plates and grown overnight at 30 °C. A 10 µL loopful of cells from each patch was used to inoculate separate 250-mL baffled Erlenmeyer flasks with 50 mL of medium consisting of 100 g/L glucose, 0.5 g/L urea, 1.5 g/L yeast extract, 0.85 g/L casamino acids, 1.7 g/L YNB without AA or ammonium sulfate, and 5.11 g/L potassium hydrogen phthalate. The pH of the flask medium started at 5.5 and declined during growth. Inoculum flasks were cultured overnight at 30 °C with constant agitation of 200 rpm in a New Brunswick I26 incubator shaker. A volume of each flask culture required to initiate a 1 L bioreactor (Dasgip, 1.2-L vessels) at a cell density of 0.5 OD_600_ was transferred to separate sterile conical tubes. Each conical tube was then brought to 50 mL with sterile deionized water and centrifuged at 3100×*g* for 3 min in an Eppendorf 5810 R centrifuge. The supernatant was decanted, and the cells were resuspended in 50 mL sterile deionized water. Inoculum was added to designated 1-L working-volume bioreactors with medium consisting of: 150 g/L glucose, 0.5 g/L (NH_4_)_2_SO_4_, 4 g/L KH_2_PO_4_, 3 g/L yeast extract, 50 mg/L Amberferm 4500, 2 g/L MgSO_4_·7H_2_O, 1 mg/L d-biotin, 12 mg/L thiamine hydrochloride, 20 mg/L ZnSO_4_·7H_2_O, 180 mg/L MnSO_4_·H_2_O, 0.03 mg/L CoCl_2_·6H_2_O, 0.2 mg/L CuSO_4_·5H_2_O, 160 mg/L Na_2_MoO_4_·2H_2_O, 800 mg/L CaCl_2_·6H_2_O, 75 mg/L FeCl_3_·6H_2_O, and 40 mg/L H_3_BO_3_. Batch process parameters were pH 3.5 automatically adjusted with 10 N sodium hydroxide, 30 °C, aeration with 0.3 vvm air, and agitation at 1000 rpm. Conditions were held constant and were such that dissolved oxygen was never limiting and always above 50%. A 10-mL sample was taken from each culture once per day. For all time-points, broth analysis was conducted via HPLC. Total dry cell weight (DCW) and total fatty acid content were measured gravimetrically by a two-phase solvent extraction. Fatty acid composition was measured by GC analysis. All results for each biological duplicate bioreactor run were averaged.

### Two-phase solvent extraction for DCW and total fatty acid content

Broth volume from each harvested culture sample was added to a separate pre-weighed 2-mL screw-cap microfuge tube (USA Scientific, 1420-8799) to achieve a cell mass between 15 and 20 mg. Samples were washed twice with deionized water and centrifuged at 21,130×*g* for 2 min. Pelleted cells were then resuspended in 200 µL of deionized water, frozen at − 80 °C for 30 min, and freeze-dried overnight. After complete drying, each tube was weighed to obtain the DCW. To each freeze-dried sample and three empty (control) microfuge tubes, 400 mg of glass beads (Sigma, G8772) and 400 µL of a 3:2, cyclopentyl methyl ether (CPME):methanol solution was added. Samples were bead-beaten with maximum agitation (BioSpec Mini-Beadbeater 8) for 2 min and cooled for 10 min to allow accumulated pressure inside the tubes to normalize before opening. After cooling, 640 µL of CPME followed by 640 µL of 10% (w/v) CaCl_2_·6H_2_O were added to each sample and vortexed. Samples were then centrifuged for 2 min at 21,130×g, creating two distinct layers. 660 µL of the top CPME layer (75% of total CPME volume) was removed and dispensed into glass vials. Samples were evaporated under compressed air until no visual solvent remained and then lyophilized overnight for total solvent removal. The remaining lipid was weighed, with any residual mass in the averaged blank samples subtracted from the mass of the experimental samples.

## Results

### Identification of genes to produce 10-methyl fatty acids

**Fig. 1:**
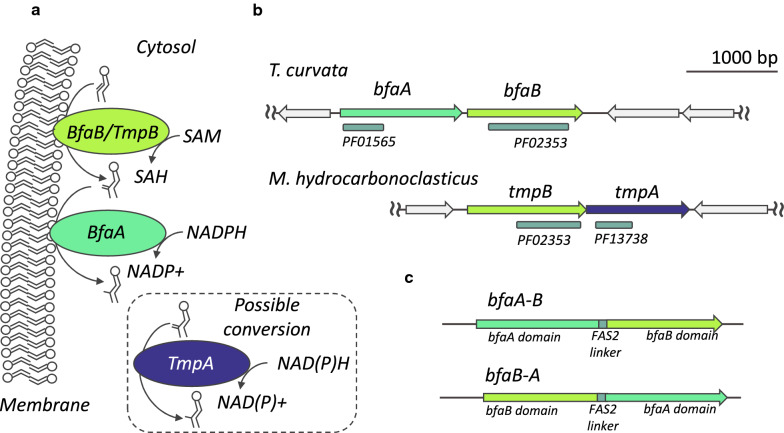
Enzymes to synthesize methyl BFAs. **a** Mechanism of 10-methyl BFA synthesis. BfaB and TmpB utilize SAM to methylate monounsaturated phospholipid-bound fatty acids in the cell membrane. BfaA uses NADPH to reduce the 10-methylene BFA to a 10-methyl BFA, while the TmpA mechanism is unknown. **b** *T. curvata bfaAB* and *M. hydrocarbonoclasticus tmpBA* gene operon topology and domain conservation are depicted. BfaB and TmpB are 31% identical and share the same protein family (PF) domain with *cfa*, which are associated with phospholipid binding and SAM binding. BfaA and TmpA both have flavoprotein domains, but they belong to different protein families and share no meaningful protein homology. **c** The engineered *bfaA-B* and *bfaB-A* gene constructs used for yeast engineering. The two protein domains are fused via a 12 amino acid linker (AGGAEGGNGGGA) derived from the *Y. lipolytica* Fas2 protein

To identify enzymes that produce 10-methyl branched fatty acids, we examined the genomes of several actinobacteria in the actinomycetales order that were reported to produce 10-methyl branched fatty acids. We searched for a gene or gene set with predicted protein domains for the two activities required for 10-methyl BFAs production: transferring a methyl group from SAM to a monounsaturated phospholipid acyl-chain resulting in a methylene branched fatty acid, and reduction to a methyl group via electrons donated by NADPH (Fig. 1a). We identified genes with over 25% amino acid identity to the *E. coli* Cfa enzyme that showed amino acid conservation in the Cfa bicarbonate ion binding site [39, 40]. Although multiple *cfa* homologs were present in the genomes of some organisms known to produce BFAs (Additional file 1: Table S1), in every instance we identified a single operon containing two genes, which were annotated in database collections as a cyclopropane-fatty-acyl-phospholipid synthase and a FAD oxidase protein (Fig. 1b, Additional file 1: Table S1, Additional file 3). The genes we identified were distinct from the *M. tuberculosis ufaA1* or *uma1* genes that have previously been ascribed to 10-methylstearic acid production. We originally termed these novel operons *tms* for their natural product, ten methyl stearate [41], but updated the name to *bfa* when we found they are homologous to the *bfaAB* gene operon from *M. chlorophenolicum* and others reported by Machida et al. [19]. To simplify expression of the two-gene operon in eukaryotic cells, we also created single-gene fusion enzymes of BfaA and BfaB connected by a flexible linker from the *Y. lipolytica* FAS2 enzyme (Fig. 1c).

Bacteria in the Desulfobacteraceae order of γ-proteobacteria also produce 10-methyl fatty acids, although the fatty acid methylation biochemistry in these bacteria is less extensively studied. Rather than 10-methylstearic acid, the primary mid-chain branched fatty acid in these bacteria is 10-methylpalmitic acid [12]. We performed a similar genome-based search in Desulfobacteraceae (Additional file 1: Table S2) and identified a two-gene operon with methyl transferase and reduction activities, which we refer to here as the *tmp* (ten methyl palmitate) gene operon. The *tmp* operon is distinct from the *bfa* operon, as the gene order differs and the BfaA protein is not homologous to the TmpA putative methylene reductase (Fig. 1b, and below).

### *bfaA* and *bfaB* expression in *E. coli*

To test whether heterologous expression of the *bfaA* and *bfaB* genes enables 10-methyl fatty acid production, we cloned the *bfaAB* operons from several donor organisms behind the constitutive *tac* promoter and expressed them in *E. coli*. In four of the eleven tested vectors, a new fatty acid was observed upon addition of exogenously fed oleic acid (Table 1). This novel fatty acid co-eluted with a 10-methylstearic acid analytical standard in GC-FID analysis, had the identical molecular weight as 10-methylstearic acid in GC–MS analysis, and was demonstrated to be fully saturated based on GC-FID analysis before and after chemical hydrogenation (data not shown). The highest conversion of oleic acid occurred with the *bfaAB* operons from *T. fusca* and *T. curvata* (Table 1). We did not further pursue the seven heterologous operons that did not demonstrate activity in our assay, possibly due to protein misfolding or other expression problems. A third *bfa* ORF of unknown function present only in some bacterial species (Additional file 1: Table S1, *bfaC*) was not necessary for branched fatty acid production (data not shown), similar to previous results [19].

### Methylene intermediate

An exo-methylene intermediate fatty acid species had previously been identified during 10-methyl fatty acid biosynthesis from whole cell extracts of *Mycobacterium phlei* [42]. To test whether recombinant expression of the putative first enzyme in the biosynthesis pathway, BfaB, would result in exo-methylene fatty acid synthesis, we cloned the *T. curvata bfaB* gene in a *Y. lipolytica* expression vector and transformed it into NS1009, a strain enriched for oleic acid production through removal of the genes for the delta-12 desaturase Fad2 and the lipase regulator Tgl3. Extracted lipid from the *bfaB* expression strain had characteristic ^13^C NMR signals for a methylene and quaternary carbon that were not present in a control extract (Additional file 2: Figure S1A). The resulting fatty acid methyl ester chromatograph contained three new peaks we assigned as 10-methylenestearic acid, 10-methylenepalmitic acid, and 8-methylenepalmitic acid (Additional file 2: Figure S1B), resulting from the methylation of three monounsaturated fatty acids present in our *Y. lipolytica* strains; C18:1 Δ9, C16:1 Δ9 and C16:1 Δ7 (the product of a single round of β-oxidation of oleic acid we and others have previously observed [23, 43, 44]).

### *bfaA* co-factor usage

*Mycobacterium phlei* whole cell extracts utilize the co-factor NADPH to reduce the methylene branch to a methyl branch in 10-methylstearic acid synthesis [15]. To evaluate whether the recombinant *T. curvata bfaA* enzyme used the same co-factor, we assayed crude cell extract from *E. coli* expressing *T. curvata bfaA* for activity on disrupted, heat inactivated recombinant *T. curvata bfaB E. coli* cells that contained 10-methylene acyl chains. We detected 10-methylstearic acid production in the presence of NADPH, but not NADH or in the absence of a reducing co-factor (Additional file 1: Table S3).

### Acyl chain substrate range

To test the acyl-chain substrate specificity of heterologously expressed *T. curvata* BfaB and BfaA enzymes, we fed exogenous unsaturated free fatty acids to a Δ*cfa E. coli* strain expressing the *bfa* pathway and an empty control vector. By comparing characteristic GC-FID retention times, we found that methylation occurred on monounsaturated acyl-chains from 14 to 20 carbons at the Δ9, Δ10, and Δ11 double bond positions (Table 2). The highest percent conversion to methylated fatty acids occurred with 16 and 18 carbon fatty acids at the Δ9 and Δ11 positions.

### *tmpBA* expression in *E. coli*

Similar to our evaluation of the *bfa* gene operon, we expressed several *tmpBA* gene operons in *E. coli* and measured branched fatty acid production (Table 1). Four of the five operons produced the branched intermediate, 10-methylenepalmitic acid. However, none of them produced 10-methylpalmitic acid, indicating the *tmpA* gene was not active in this assay. Intriguingly, TmpA does not resemble BfaA by domain homology (Fig. 1), but has similarity to anaerobic archaeal geranylgeranyl reductases that reduce the double bonds in isoprenoid-derived membrane lipids [45]. It is possible that TmpA is oxygen sensitive or requires an electron carrier not present in our *E. coli* cells. We did not pursue further *tmpA* characterization because our intended yeast host organisms produce fatty acids primarily under aerobic conditions.

The acyl-chain substrate specificity of the heterologously expressed *tmp* operons from *M. hydrocarbonoclasticus* and *T. halophila* were evaluated in a Δ*cfa E. coli* strain. Methylene branched fatty acids were detected for monounsaturated acyl-chains from 14 to 18 carbons at the Δ9, Δ10, and Δ11 double bond positions (Table 2). The highest percent conversion to methylated fatty acids occurred with 14 and 16 carbon fatty acids unsaturated at the Δ9 position.

### Production of 10-methylene BFAs in yeast

**Fig. 2:**
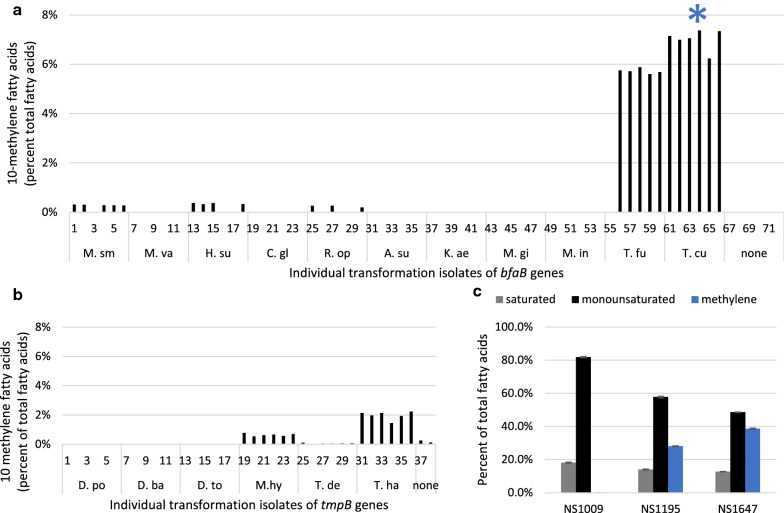
Engineering production of 10-methylene BFAs in *Y. lipolytica.* To measure fatty acid species, plate lipid accumulation assays were carried out and individual fatty acids species were quantified by GC-FID and reported as the percentage of total C16 and C18 fatty acid species. **a** Strain NS1009 was transformed with replicating plasmids containing the indicated *bfaB* genes from *M. smegmatis* (pNC915), *M. vanbaaleni* (pNC917), *H. subflava* (pNC918), *C. glyciniphilum* (pNC919), *R. opacus* (pNC920), *A. subbeticus* (pNC923), *K. aerolata* (pNC924), *M. gilvum* (pNC925), *M. indicus* (pNS926), *T. fusca* (pNC921), *T curvata* (pNC922). C10-methylene fatty acids were measured. * Indicates NS1117. **b** Strain NS1009 was transformed with integrating constructs expressing *tmpB* genes from *D. postgatei* (pNC996), *D. balticum* (pNC998), *D. toluoica* (pNC1000), *M. hydrocarbonclasticus* (pNC1002), *T. denitrificans* (pNC1004), *T. halophila* (pNC1006). The sum of all C8 and C10-methylene fatty acids was measured. **c** Fatty acid profiles were measured for NS1009 and strains containing one (NS1195) and two (NS1647) integrated copies of the *T. curvata bfaB* gene. The sum of all saturated, monounsaturated, and C8 and C10-methylene fatty acids was measured, and the mean and range of samples from duplicate biological experiments is shown

To produce 10-methylene fatty acids in yeast, eleven *bfaB* candidate genes were expressed in *Y. lipolytica*. The *bfaB* genes were subcloned into *Y. lipolytica* expression vectors and transformed into NS1009. Multiple individual transformants for each *bfaB* gene were selected to undergo a 4-day lipid accumulation assay in 96-well plates, after which 10-methylene fatty acids were measured. Both 10-methylenepalmitic acid and 10-methylenestearic acid were detected at varying levels in individually isolated strains, and for simplicity we report the total levels of all C16 and C18 10-methylene fatty acids (Fig. 2a). Low levels of 10-methylene BFAs were produced by BfaB from *M. smegmatis*, *H. subflava* and *R. opacus,* while higher production was measured for BfaB from *T. fusca* and *T. curvata* (Fig. 2a). The isolate producing the most 10-methylene fatty acids in this initial screen was strain NS1117 expressing *T. curvata bfaB* on a replicating plasmid. The activity of six TmpB enzymes were similarly assayed in *Y. lipolytica*, and the genes from *M. hydrocarbonoclasticus* and *T. halophila* produced low levels of 10-methylene fatty acids (Fig. 2b). These two genes, plus the *tmpB* gene of *D. balticum* showed higher activity in *S. cerevisiae* when expressed from a high-copy plasmid (Additional file 2: Figure S2), suggesting that the enzymes are active in yeast, but activity is limited in *Y. lipolytica*.

We selected the *T. curvata bfaB* gene for further BFA production in *Y. lipolytica*. To construct stable strains, we chromosomally integrated the *T. curvata bfaB* gene in subsequent constructions. We utilized a random integration approach, which allowed screening of individual isolates with different total expression levels, to assess the range of phenotypes associated with overexpression of each gene. When we screened 96 individual integration colonies for the highest 10-methylene BFA content, we isolated strain NS1195, which demonstrated 10-methylene fatty acid levels up to 28% of total fatty acids in our plate-based assay (Fig. 2c). Consistent with the idea that gene expression was limiting, integrating a second copy of *T. curvata bfaB* with selection from 96 colonies yielded strain NS1647, which had a 10-methylene BFA content of 39% of total fatty acids (Fig. 2c). With each integration we saw a concomitant decrease in monounsaturated fatty acids, the substrate for the BfaB enzyme. Strains producing high levels of 10-methylene BFAs exhibited decreased growth in *Y. lipolytica* (data not shown, discussed further below), similar to observations from expression of Bfa enzymes in cyanobacteria [20].

### Production of 10-methyl BFAs in yeast

**Fig. 3:**
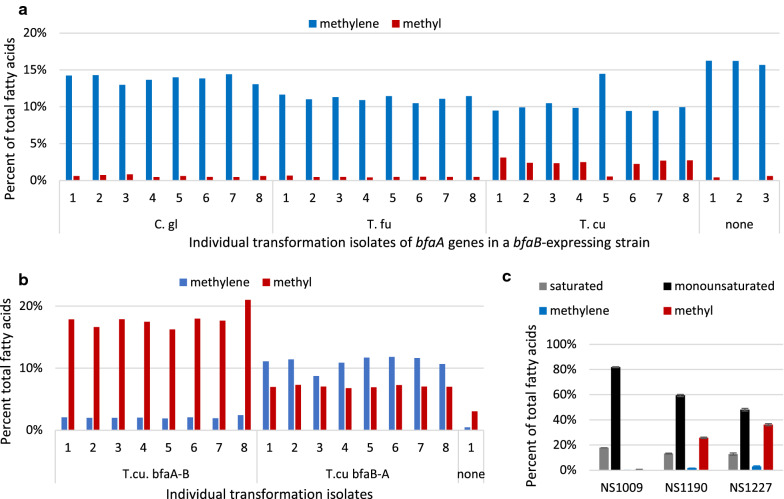
Engineering production of 10-methyl BFAs in *Y. lipolytica*. To measure fatty acid species, plate lipid accumulation assays were carried out and individual fatty acids species were quantified by GC-FID and reported as the percentage of total C16 and C18 fatty acid species. **a** NS1117 expressing the *T. curvata bfaB* gene was transformed with CEN plasmids expressing *bfaA* genes from *C. glutamicum* (pNC1024), *T. fusca* (pNC1025) and *T. curvata* (pNC984) and a no plasmid control. C8- and C10-methylene (blue) and methyl (red) fatty acids were measured. Signal in the no plasmid control represents GC background signal in this experiment. **b** NS1009 was transformed with integrating constructs expressing *T. curvata bfaA-B* and *bfaB-A* fusion proteins. C8- and C10-methylene (blue) and methyl (red) fatty acids were measured. **c** Saturated, monounsaturated, 8- and 10-methylene, and 8- and 10-methyl fatty acid species were measured for NS1009 and strains expressing one (NS1190) or two (NS1227) integrated copies of *T. curvata bfaA-B.* The mean and range of samples from duplicate biological experiments is shown

To produce 10-methyl BFAs, the 10-methylene BFA-producing strain NS1117 was transformed with various *bfaA* and *tmpA* genes. No activity was detected for any *tmpA* gene (data not shown), similar to our results in *E. coli*. In contrast, *Y. lipolytica* strains expressing the *C. glutamicum*, *T. fusca* and *T. curvata bfaA* enzymes all exhibited some production of 10-methyl fatty acids (Fig. 3a). The strain expressing both *T. curvata bfaA* and *T. curvata bfaB* had the highest conversion rate, suggesting either that *T. curvata* BfaA has the most activity in yeast, or there is a direct interaction between the BfaA and BfaB proteins from the same organism that serves to increases conversion of BFAs. However, all strain isolates expressing both *T. curvata bfaA* and *T. curvata bfaB* showed inefficient production of 10-methyl BFAs, with the majority of accumulated BFAs remaining in the methylene form (Fig. 3a).

To increase the conversion of 10-methylene BFAs to 10-methyl BFAs, we linked the *T. curvata bfaA* and *bfaB* genes in frame to produce the single fusion proteins, BfaA-B and BfaB-A (Fig. 1c). When expressed in *Y. lipolytica*, the BfaA-B fusion protein significantly increased 10-methyl BFA production so that ~ 90% of BFAs are in the fully saturated form (Fig. 3b). The BfaB-A fusion did not work as efficiently in *Y. lipolytica,* where it produced more 10-methyl BFAs than the unjoined genes, but high levels of methylene BFAs remained (Fig. 3b). Both BfaA-B and BfaB-A efficiently produced 10-methyl BFAs when expressed from the *Y. lipolytica TEF1* promoter on a high-copy (2μ) plasmid in *S. cerevisiae* (Additional file 2: Figure S3), indicating the lack of BfaA reduction activity for *bfaB-A* expressing *Y. lipolytica* strains is likely due to inefficient read-through in that strain, not defective enzyme activity in the fusion protein.

To further enhance 10-methyl BFA production, we added a second copy of *bfaA-B* to our best *Y. lipolytica* BFA production strain. Among the individual isolates assayed, two had very high levels of 10-methyl BFA production, exceeding 50% of total fatty acids (data not shown). However, these two isolates also exhibited significant growth and lipid accumulation defects (data not shown), precluding their use in large-scale production. Throughout our studies, we repeatedly observed an association between very high BFA content and poor growth. We hypothesize that these growth defects are not accidental mutations arising from random integration events, but rather the specific effect of high levels of BfaB or BfaA-B activity. Therefore, we chose to proceed with the analysis of an isolate, NS1227, that demonstrated lower BFA composition (36%), but better growth in our lipid accumulation conditions (Fig. 3c).

### Secretion of BFAs in *S. cerevisiae*

**Fig. 4:**
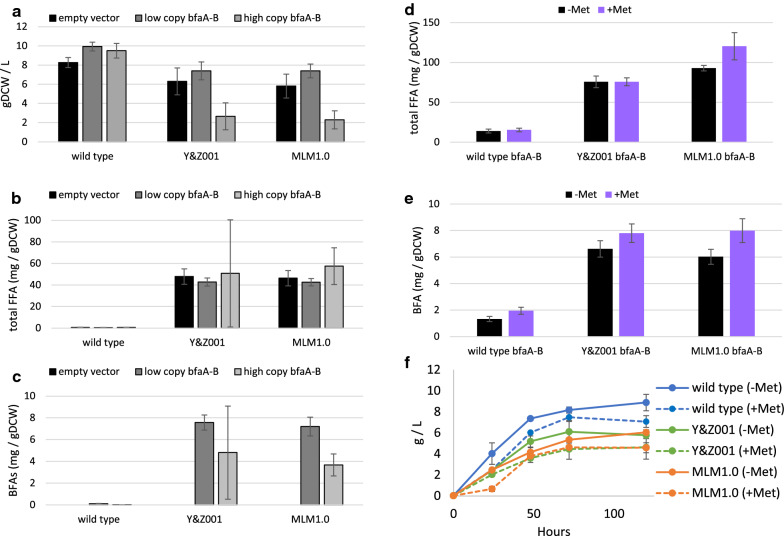
Expression of *bfaA-B* in *S. cerevisiae*. For all data the average and standard deviation of 3 independent experiments are shown. **a** Comparison of total dry cell weight (DCW) for wild-type (CEN.PK), Y&Z001 and MLM1.0 with empty vector (pRS416), low-copy *bfaA-B* (in pRS416) or high-copy *bfaA-B* (in pRS426). OD_600_ was measure after 72 h growth in minimal medium and converted using the measured constants for 1 OD for wild-type (0.687 ± 0.026), Y&Z001 (0.634 ± 0.017), and MLM1.0 (0.632 ± 0.047). **b** Comparison of total FFAs in the experiment shown in **a** was normalized to DCW. **c** Comparison of total BFA levels from the experiment in **a** normalized to the DCW. **d** Total FFAs and OD were measured for three *S. cerevisiae* strains expressing p416 *bfaA-B* grown in the absence or presence of 1.3 g/L methionine for 72 h in minimal medium. OD was converted to DCW using the same conversion constants as in **a** from. **e** Measurement of C16 and C18 BFAs from the experiment in **d** are shown. **f** Growth profile from the experiment shown in **d** and **e**

We tested whether secreting free fatty acids (FFAs) could increase the production and turnover of BFAs. We initially tested FFA secretion in *Y. lipolytica* similar to previous studies [26], however the secretion strains constructed in the YB-392 background grew poorly and were not appropriate for industrial fermentation. Therefore, we utilized *S. cerevisiae* strains engineered for enhanced free fatty acid production based on preventing fatty acid activation and degradation through deletion of *FAA1*, *FAA4* and *POX1* [24, 25]*.* In addition, Y&Z001 carries a synthetic acetyl-CoA and malonyl-CoA overproduction pathway [24], whereas MLM1.0 has upregulated phospholipid synthesis and turnover [25]. Although these strains are engineered to prevent fatty acid activation and degradation, we have previously observed that secreted FFAs seem to originate from phospholipid acyl chains because they persist even when other pools of cellular fatty acids have been depleted and their production is correlated with increasing phospholipid levels [25]. Using *S. cerevisiae* constructs expressing codon-optimized *bfaA-B* with the *S. cerevisiae TEF1* promoter, we found that the wild-type CEN.PK strain grew well with single-copy or high-copy *bfaA-B* plasmids (Fig. 4a), although this low lipid strain is not appropriate for large-scale production. In both FFA production strains, high-copy *bfaA-B* expression caused highly variable growth, total free fatty acid (FFA) and BFA production. However, low copy expression was tolerated well (Fig. 4a–c). Y&Z001 and MLM1.0 strains expressing *bfaA-B* produced 56 and 53 mg BFA/L, which corresponded to 7.6 and 7.2 mg BFA/gDCW, respectively, in shake-flask assays (Fig. 4c).

We next tested whether BFA production strains have limited levels of the methyl donor, SAM, which is also required for a variety of cellular methylation reactions [46]. The most prevalent essential pathway utilizing SAM is the production of phosphatidylcholine (PC) from phosphatidylethanolamine (PE) [37]. We estimate that the wild-type CEN.PK strain requires 23–40 μmol SAM/g dry cell weight (DCW), and the MLM1.0 increased lipid strain [25] would require 73–119 μmol SAM/g DCW. We tested whether increasing intracellular SAM levels could improve growth and BFA production. SAM is not stable in growth media, however, previous studies have established that methionine supplementation increases intracellular SAM levels in *S. cerevisiae* [47], but with toxic effects [48, 49]. We chose to supplement with 1.3 g/L methionine to maximize SAM levels while minimizing growth effects. We compared total FFA and BFA production in the absence or presence of methionine in shake-flask fermentation experiments. The MLM1.0 strain that over-produces phospholipids showed slightly increased total FFA production (Students *t*-test *p* = 0.103, standard deviations do not overlap) with methionine supplementation (Fig. 4d), consistent with the role of SAM in PC production. All strains showed some increase in BFA production with methionine (Fig. 4e), however, the results were not highly significant (students *t* test *p* values of 0.122 for wild type, 0.096 for Y&Z001 and 0.042 for MLM1.0). Methionine supplementation decreased the growth of all strains (Fig. 4f). With methionine addition, Y&Z001 and MLM1.0 strains both produced approximately 8 mg total BFA/gDCW, a significant improvement over the wild-type strain that produced 2 mg BFA/gDCW in this assay. Methionine supplementation of *Y. lipolytica* strains did not improve BFA production (data not shown), although it has been reported that methionine addition does not increase SAM levels in this organism [50], due to unique sulfur metabolism pathways [51]. These results suggest that SAM levels may become limiting when BFA production occurs at the same time as increased phospholipid synthesis, such as during cell-growth.

One hypothesis for the poor growth observed in strains producing very high levels of BFAs is that BFAs themselves are toxic to cells, possibly due to changes in membrane structure and fluidity similar to those seen with cyclopropanated phospholipids [52]. To understand where BFAs are produced in the cells, we examined BfaA-B localization in live *S. cerevisiae* by linking the protein to GFP. In bacteria, the Bfa enzymes are thought to act at the plasma membrane although they are not integral membrane proteins (Fig. 1a), similar to cfa synthase [40]. In the Y&Z001 strain both GFP-BfaA-B and BfaA-B-GFP fusions localized to the plasma membrane and punctate structures resembling lipid bodies (Additional file 2: Figure S4A). The localization of the GFP fusion proteins to lipid bodies was confirmed using the neutral lipid dye Nile Red in a strain engineered for increased lipid droplets [32] (Additional file 2: Figure S4B). This staining pattern suggests that BFAs are likely produced in the yeast plasma membrane and/or the lipid body membrane, and we have confirmed that they accumulate to high levels in TAG oil in *Y. lipolytica* (data not shown).

### Production of 10-methylene and 10-methyl BFAs in bioreactors

**Fig. 5:**
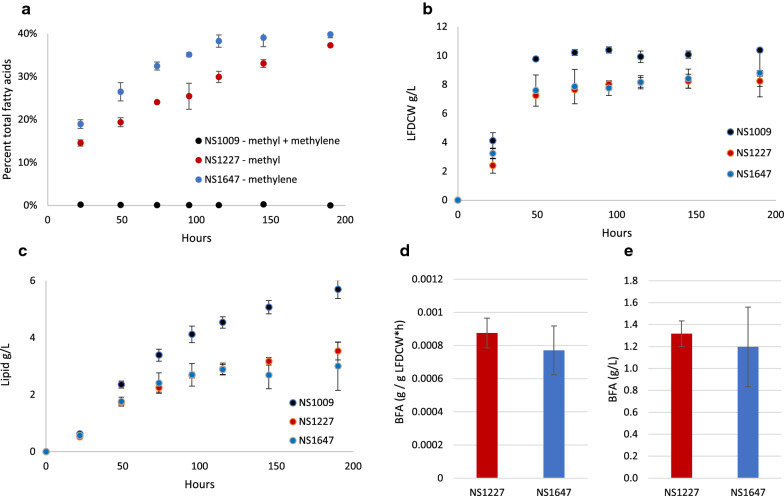
Production of BFAs in *Y. lipolytica* in bioreactors*.* The average and range of each value measured in two independent experiments for each strain are shown. **a** 10-methyl and 10-methylene fatty acid production was measured by GC throughout the fermentation time course and expressed as the percent of total fatty acids. **b** Growth of the cells was monitored by measuring the cell mass produced after extraction of soluble lipid. **c** Total lipid production was measured by isolating and weighing the extracted lipid from each sample. **d** The cell-specific productivity was calculated for 10-methyl BFAs (NS1227) and 10-methylene BFAs (NS1647). **e** Final titer of BFAs was calculated for 10-methyl BFAs (NS1227) and 10-methylene BFAs (NS1647)

To better characterize BFA production, we performed 1 L batch fermentations for the strains that had the best combination of growth, total lipid production and BFA production: *Y. lipolytica* strains NS1227 (2 copies of *T. curvata bfaA-B*) and NS1647 (2 copies of *T. curvata bfaB*), using the parental strain NS1009 as a control. Two independent fermentations were carried out for each strain and the average results were reported. Of total cellular fatty acids produced by 190 h, NS1647 accumulated 40% 10-methylene fatty acids and NS1227 accumulated 37% 10-methyl fatty acids (Fig. 5a). However, as noted in our plate assays, cells producing 10-methylene and 10-methyl BFAs showed a decreased rate of cell mass accumulation when compared to NS1009 (Fig. 5b) and consumed proportionately less glucose (Additional file 2: Figure S5). The decreased cell weight can be explained both by the slower cell division observed at our earliest sample time (Fig. 5b first time point), as well as lower total lipid levels accumulated in stationary phase in the BFA-producing strains (Fig. 5c). NS1647 accumulated 10-methylene BFAs faster than NS1227 accumulated 10-methyl fatty acids (Fig. 5a), likely due to different levels of expression of the randomly integrated gene copies. Additionally, the BfaA-B protein is significantly larger (887 amino acids) than BfaB alone (420 amino acids), which might decrease the protein accumulation rate through slower translation or folding. To compare the two BFA-producing strains, we calculated cell-specific productivity to normalize the BFA levels to the lipid-free dry cell weight (LFDCW) for each strain. NS1227 and NS1647 showed no significant difference in 10-methyl or 10-methylene BFA production, with respective average rates of 8.8e−4 and 7.7e−4 g BFA per g of LFDCW per hour (Fig. 5d, Additional file 2: Figure S5). The total BFA content of both strains was also similar; NS1227 produced BFAs at 11.3% of the total cell mass with a titer of 1.3 g/L 10-methyl fatty acids, and NS1647 produced BFAs at 10% of the total cell mass with a titer of 1.2 g/L 10-methylene fatty acids (Fig. 5e).

## Discussion

We demonstrate here production of 10-methylene and 10-methyl branched fatty acids. 10-Methylene fatty acids are produced in yeast from Δ9–10 monounsaturated C16 and C18 fatty acids using methyltransferases from multiple species of both mycobacteria (*bfaB*) and γ-proteobacteria (*tmpB*). 10-Methyl fatty acids were observed when both *bfaA* and *bfaB* genes were expressed, and production was most efficient when the two genes were fused together as *bfaA-B*. Overexpression of the *T. curvata* BfaB or the BfaA-B fusion protein in *Y. lipolytica* enabled the production 10-methylene and 10-methyl BFAs, respectively, with a titer of at least 1.2 g/L in a 1 L batch fermentation process.

Although 10-methyl BFAs were isolated from mycobacterium in 1943 [53], the enzymes responsible for the two-step synthesis of 10-methylstearic acid from oleic acid were not reported until recently [19]. The BfaA and BfaB enzymes identified here are homologous to those identified by Machida et al. [19]. In addition, we isolated a second set of genes from γ-proteobacteria that likely carry out a similar methylation reaction on palmitic acid in vivo, to produce 10-methylpalmitatic acid (tmp). The *tmpB* genes showed robust methylation activity in *S. cerevisiae*, but we were unable to observe activity for the *tmpA* genes in aerobically growing yeasts or *E. coli*. We found that the *T. curvata * BfaB and the *M. hydrocarbonoclasticus* and *T. halophila *TmpB can methylate a variety of C14–C20 fatty acids that are monounsaturated at the Δ9, Δ10, and Δ11 positions, with the BfaB enzyme preferring C16–C18 fatty acids and TmpB preferring C14–C16 fatty acids. Consistently, we found TmpB activity was robust in *S. cerevisiae*, where C16 fatty acids are more prevalent compared to our *Y. lipolytica* strain, which produces predominantly C18 fatty acids [23]. Both *bfa* and *tmp* two-gene operons were identified by homology searches for an ORF homologous to the *E. coli cfa* gene with an adjacent gene containing reductase activity. We believe that the *bfa* and *tmp* gene sets were independently evolved as evidenced by the lack of homology between the BfaA and TmpA proteins, the opposite gene order in each family, and the distant evolutionary relationship of the bacterial species from which they were isolated.

We consistently observed that production of large amounts of BFAs in yeast resulted in decreased growth and lipid accumulation. Overexpression of *bfaB* alone was sufficient to affect growth and lipid, indicating that neither *bfaA* nor the 10-methyl product is required for the effect. This finding is in agreement with the effects of *M. chlorophenolicum bfaA* and *bfaB* overexpression in cyanobacteria [20]. The methylation reaction by BfaB utilizes SAM as the methyl donor, and one possibility is that the co-factor may be limited under *bfaB* overexpression conditions, competing with essential cellular functions that also require SAM. Our results in *S. cerevisiae* suggested that SAM can be limiting for phospholipid and BFA production, and it is possible that modifications in culture conditions and genes in the SAM pathway could improve BFA production in yeast. Alternatively, and non-exclusively, the production of high levels of methylene or methyl BFAs in the cell or organelle membranes may be harmful to the cells, as the methyl addition is expected to alter membrane fluidity and structure [54]. Increasing the turnover of BFAs from cell membranes may be important for decreasing the effects on cell proliferation.

In this study, we have explored two routes to accumulate BFAs, through secretion of FFAs or accumulation in TAGs. Although we do not know how BFAs are mobilized after synthesis in the plasma membrane, we have demonstrated that BFAs are secreted by *S. cerevisiae* cells lacking FFA activation and degradation pathways. The titers of total fatty acids, including BFAs, were low in this system, indicating that improvements are necessary to render this production method commercially feasible. The advantages of the secretion system would be to simplify FFA recovery and purification, and to enable cost-saving continuous fermentation. As an alternative, we have also shown that BFAs accumulate efficiently in TAGs in *Y. lipolytica*, where we demonstrated the highest composition yet reported for 10-methyl BFA production, at 37% of total fatty acids. Previous studies reported BFA compositions of 4.1% in *Synechocystis* and 14% in the native producer *M. chlorophenolicum* [20]. Total DCW contents were not calculated in the previous studies, but natural lipid contents relative to DCW reported for *Synechocystis* are 10–20% [55, 56], indicating the BFA content is approximately 0.4–0.8% of DCW. In the current study, the strains expressing BFA genes in *Y. lipolytica* had vastly increased BFA contents of 10–11.3% DCW. It is possible that BFA content and productivity can be further improved by combining *T. curvata bfaA-B* expression with genetic modifications previously demonstrated to increase lipid yield in *Y. lipolytica* [22]. Additionally, Imatoukene et al. demonstrated improved cyclopropane fatty acid production in *Y. lipolytica* through a combination of genetic and process improvements [57], and a similar approach could be applied to 10-methyl BFA production.

## Conclusions

BFAs are of interest due to their potential applications in a wide variety of commercial products. In *Y. lipolytica* we produced primarily 10-methylstearic acid, a fully saturated long-chain fatty acid with high viscosity, low pour point and high oxidative stability. We also produced 10-methylenestearic acid, an unusual fatty acid with potential for chemical derivatization to novel oleochemicals. We identified genes capable of producing mono-methyl derivatives of a variety of other fatty acid chain-lengths and positions, which could be tailored to specific applications. The yeasts *S. cerevisiae* and *Y. lipolytica* are promising hosts for the further development of BFA production processes for these unique and valuable fatty acids.

## Supplementary Information

**Additional file 1: **Tables.

**Additional file 2: **Figures.

**Additional file 3. **Sequences and alignments.

## Data Availability

All data generated or analyzed during this study are included in this published article and its Additional files.
