# Supplementary material for: Production of 10-methyl branched fatty acids in yeast
Source: Biotechnol Biofuels. 2021 Jan 7;14:12. doi: 10.1186/s13068-020-01863-0 (PMC7791843; doi:10.1186/s13068-020-01863-0)
Supplement: Supplementary file 1 — Additional file 1: Tables. [file 13068_2020_1863_MOESM1_ESM.docx]

**Table S1.** Bacteria used to identify the bfa gene operon

| Microorganism | 10Me18 fatty acids | Number of *cfa* synthase homologs | *bfa* operon present | *bfaB* genome locus tag | *bfaA* genome locus tag | *bfaC* genome locus tag | Reference |
| --- | --- | --- | --- | --- | --- | --- | --- |
| *Mycobacterium smegmatis* mc2 155 | yes | 7 | yes | MSMEI_RS30765 | MSMEI_RS30760 | - | Etienne 2005 |
| *Corynebacterium glyciniphilium* AJ3170 | yes | 4 | yes | CGLY_02225 | CGLY_02220 | CGLY_02230 | Al-Dilaimi 2015 |
| *Escherichia coli* MG1655 | no | 1 | no | - | - | - | Grogan 1997 |
| *Mycobacterium tuberculosis* H37Rv | yes | 4 | yes | Rv3720 | Rv3719 | - | Phetsuksiri 2003 |
| *Mycobacterium phlei* RIVM601174 | yes | 10 | yes | MPHLEI_RS13770 | MPHLEI_RS13765 | - | Campbell 1969 |
| *Thermomonospora curvata* DSM 43183 | yes | 3 | yes | TCUR_RS07780 | TCUR_RS07775 | - | Zhang 1998 |
| *Rhodococcus opacus* PD630 | yes | 7 | yes | PD630_RS02880 | PD630_RS02885 | - | Waltermann 2000 |
| *Actinomyces israelii* DSM 43320 | no | 1 | no | - | - | - | Kroppenstedt 1985 |
| *Clostridium acetobutylicum* ATCC 824 | no | 1 | no | - | - | - | Grogan 1997 |
| *Corynebacterium renale* DSM 20298 | no | 0 | no | - | - | - | Kroppenstedt 1985 |
| *Streptomyces verne* DSM 40079 | no | 0 | no | - | - | - | Kroppenstedt 1985 |

This table was assembled with genomic sequence information collected from Genbank and Metacyc databases, and the presence of 10-methyl branched fatty acids from the included references.

**Table S2.** Bacteria used to identify the tmp gene operon

| Microorganism | 10Me16 fatty acids | Number of *cfa* synthase homologs | *tmp* operon | *tmpB* genome locus tag | *tmpA* genome locus tag | Reference |
| --- | --- | --- | --- | --- | --- | --- |
| *Desulfobacter curvatus* DSM 3379 | yes | 2 | yes | B147_RS0124955 | B147_RS0124950 | Kohring 1994 |
| *Desulfobacter postgatei* 2ac9 | yes | 2 | yes | DESPODRAFT_RS04665 | DESPODRAFT_RS04670 | Dowling 1986 |
| *Desulfobacula toluolica* Tol2 | yes | 3 | yes | TOL2_C28310 | TOL2_C28300 | Kuever 2001 |
| *Desulfotomaculum acetoxidans* DSM 771 | no | 0 | no | - | - | Dowling 1986 |
| *Desulfuromonas acetoxidans* DSM 684 | no | 0 | no | - | - | Dowling 1986 |
| *Escherichia coli* MG1655 | no | 1 | no | - | - | Taylor 1978 |
| *Marinobacter hydrocarbonoclasticus* ATCC 49840 | yes | 2 | yes | MARHY3375 | MARHY3376 | Marquez 2005 |
| *Pseudomonas putida* KT2440 | no | 2 | no | - | - | Pini 2011 |
| *Thiohalospira halophila* DSM 15071 | yes | 2 | yes | SAMN05660831_00818 | SAMN05660831_00819 | Sorokin 2008 |

This table was assembled with genomic sequence information collected from Genbank and Metacyc databases, and the presence of 10-methyl branched fatty acids from the included references.

**Table S3.** *T. curvata bfaA* co-factor usage.

| *E. coli* (*Δcfa* background) cell free extract | co-factor | relative 10-Methyl C16+10-Methyl C18 peak area | SD |
| --- | --- | --- | --- |
| *T. curvata bfaA* | NADPH | 0.059 | 0.003 |
| *T. curvata bfaA* | NADH | 0 | 0 |
| *T. curvata bfaA* | none | 0 | 0 |
| empty vector | NADPH | 0 | 0 |
| empty vector | NADH | 0 | 0 |
| empty vector | none | 0 | 0 |
| none | NADPH | 0 | 0 |
| none | NADH | 0 | 0 |
| none | none | 0 | 0 |

Cell free extracts were incubated with assay components for 16 hours as described in the Methods section. Relative 10-Methyl C16+10-Methyl C18 peak area is the sum of the 10-methyl BFA gas chromatography peak areas divided by the total fatty acid GC area sum from the assay.

**Table S4**. Plasmids used in this study

| Plasmid number | host | backbone | Expression gene | Expression (Integrating (I), Plasmid (P),  ARS/CEN (C), 2-micron) |
| --- | --- | --- | --- | --- |
| pNC53 | *E. coli* | Amp, ScURA3, pTAC,trpT’ | none | P |
| pNC704 | *E. coli* | pNC53 | *Mycobacterium smegmatis bfa* operon | P |
| pNC721 | *E. coli* | pNC53 | *Mycobacterium vanbaaleni bfa* operon | P |
| pNC755 | *E. coli* | pNC53 | *Amycolicicoccus subflavus bfa* operon | P |
| pNC757 | *E. coli* | pNC53 | *Corynebacterium glyciniphilum bfa* operon | P |
| pNC904 | *E. coli* | pNC53 | *Rhodococcus opacus bfa* operon | P |
| pNC905 | *E. coli* | pNC53 | *Thermobifida fusca bfa* operon | P |
| pNC906 | *E. coli* | pNC53 | *Thermomonospora curvata bfa* operon | P |
| pNC907 | *E. coli* | pNC53 | *Corynebacterium glutamicum bfa* operon | P |
| pNC908 | *E. coli* | pNC53 | *Agromyces subbeticus bfa* operon | P |
| pNC910 | *E. coli* | pNC53 | *Mycobacterium gilvum bfa* operon | P |
| pNC911 | *E. coli* | pNC53 | *Mycobacterium sp. indicus bfa* operon | P |
| pNC915 | yeast | NAT, ARS68/CEN1-1, pYlTEF, AaCYC1ter | *Mycobacterium smegmatis bfaB* | C |
| pNC917 | yeast | NAT, ARS68/CEN1-1, pYlTEF, AaCYC1ter | *Mycobacterium vanbaaleni bfaB* | C |
| pNC918 | yeast | NAT, ARS68/CEN1-1, pYlTEF, AaCYC1ter | *Amycolicicoccus subflavus bfaB* | C |
| pNC919 | yeast | NAT, ARS68/CEN1-1, pYlTEF, AaCYC1ter | *Corynebacterium glyciniphilum bfaB* | C |
| pNC920 | yeast | NAT, ARS68/CEN1-1, pYlTEF, AaCYC1ter | *Rhodococcus opacus bfaB* | C |
| pNC921 | yeast | NAT, ARS68/CEN1-1, pYlTEF, AaCYC1ter | *Thermobifida fusca bfaB* | C |
| pNC922 | yeast | NAT, ARS68/CEN1-1, pYlTEF, AaCYC1ter | *Thermomonospora curvata bfaB* | C |
| pNC923 | yeast | NAT, ARS68/CEN1-1, pYlTEF, AaCYC1ter | *Agromyces subbeticus bfaB* | C |
| pNC924 | yeast | NAT, ARS68/CEN1-1, pYlTEF, AaCYC1ter | *Knoellia aerolata bfaB* | C |
| pNC925 | yeast | NAT, ARS68/CEN1-1, pYlTEF, AaCYC1ter | *Mycobacterium gilvum bfaB* | C |
| pNC926 | yeast | NAT, ARS68/CEN1-1, pYlTEF, AaCYC1ter | *Mycobacterium sp. indicus bfaB* | C |
| pNC983 | yeast | NAT, ARS68/CEN1-1, pYlTEF, AaCYC1ter | *Thermobifida fusca bfaA* | C |
| pNC984 | yeast | NAT, ARS68/CEN1-1, pYlTEF, AaCYC1ter | *Thermomonospora curvata bfaA* | C |
| pNC1024 | yeast | NAT, ARS68/CEN1-1, pYlTEF, AaCYC1ter | *Corynebacterium glyciniphilum bfaA* | C |
| pNC1034 | yeast | hsvTDK, NAT, pYlTEF, AaADH1ter | *Thermomonospora curvata bfaB* | I + 2-micron |
| pNC1067 | yeast | hsvTDK, HYG, pYlTEF, AaADH1ter | *Thermomonospora curvata bfaA-B* | I + 2-micron |
| pNC1071 | *E. coli* | pNC53 | *Desulfobacter postgatei tmp operon* | P |
| pNC1072 | *E. coli* | pNC53 | *Desulfobacter balticum tmp operon* | P |
| pNC1073 | *E. coli* | pNC53 | *Desulfobacula toluolica tmp operon* | P |
| pNC1074 | *E. coli* | pNC53 | *Marinobacter hydrocarbonoclasticus tmp operon* | P |
| pNC1076 | *E. coli* | pNC53 | *Thiohalospira halophila tmp operon* | P |
| p416-bfaAB | yeast | URA3, ARS/CEN, p416TEF | *Thermomonospora curvata bfaA-B* codon-optmized for *S. cerevisiae* | P |
| p426-bfaAB | yeast | URA3, 2micron, p416TEF | *Thermomonospora curvata bfaA-B* codon-optmized for *S. cerevisiae* | 2-micron |

**Table S5**. Yeast and bacterial strains used in this study

| Strain number | Organism | Genotype (all deletions and insertions unmarked in the final strain unless indicated) |
| --- | --- | --- |
| NS20 | *S. cerevisiae INVSc1* | *MATa his3D1 leu2 trp1-289 ura3-52 MATalpha his3D1 leu2 trp1-289 ura3-52* |
| NS1009 | *Y. lipolytica YB-392* | *tgl3Δ, fad2Δ* |
| NS1117 | *Y. lipolytica* | *tgl3Δ, fad2Δ, T. curvata bfaB::NAT CEN plasmid* |
| NS1138 | *E. coli CGSC 9407* | *Δcfa kanR (JW1653-1 Keio collection)* |
| NS1161 | *E. coli* | *Δcfa kanR, empty vector pNC53 ampR* |
| NS1162 | *E. coli* | *Δcfa kanR, T. curvata tmsAB, ampR* |
| NS1163 | *E. coli* | *Δcfa kanR T. curvata tmsB ampR* |
| NS1164 | *E. coli* | *Δcfa kanR T. curvata tmsA ampR* |
| NS1190 | *Y. lipolytica* | *tgl3Δ, fad2Δ, T. curvata bfaA-B* |
| NS1195 | *Y. lipolytica* | *tgl3Δ, fad2Δ, T. curvata bfaB* |
| NS1227 | *Y. lipolytica* | *tgl3Δ, fad2Δ, 2copies T. curvata bfaA-B* |
| NS1237 | *E. coli* | *Δcfa kanR, M. hydrocarbonoclasticus tmpBA, ampR* |
| NS1238 | *E. coli* | *Δcfa kanR, T. halophila tmpBA, ampR* |
| NS1647 | *Y. lipolytica* | *tgl3Δ, fad2Δ, 2copies T. curvata bfaB* |
| CEN.PK113-5D | *S. cerevisiae CEN.PK113* | *MATa ura3-52 TRP1 LEU2 HIS3* |
| Y&Z001 | *S. cerevisiae CEN.PK113* | *MATa ura3-52 TRP1 LEU2 HIS3 Δhfd1, Δpox1, Δfaa1, Δfaa4, MmACL, RtME, CTP1, ’MDH3, ’tesA, RtFAS1, RtFAS2* |
| MLM1.0 | *S. cerevisiae CEN.PK113* | *MATa ura3-52 TRP1 LEU2 HIS3* can1∆::cas9-natNT2 *faa1Δ faa4Δ pox1Δ dga1Δ lro1Δ are1Δ dpp1Δ lpp1Δ pah1Δ* |
| RF07 | *S. cerevisiae CEN.PK113* | *MATa ura3-52 TRP1 LEU2 HIS3* can1∆::cas9-natNT2 *ACC1**PAH1 DGA1 tgl3Δ tgl4Δ tgl5Δ* |
