# Supplementary material for: Production of 10-methyl branched fatty acids in yeast
Source: Biotechnol Biofuels. 2021 Jan 7;14:12. doi: 10.1186/s13068-020-01863-0 (PMC7791843; doi:10.1186/s13068-020-01863-0)
Supplement: Supplementary file 2 — Additional file 2: Figures. [file 13068_2020_1863_MOESM2_ESM.docx]

**Figure S1**. *tmpB* produces 10-methylene palmitate and 10-methylene stearate in *Yarrowia lipolytica*


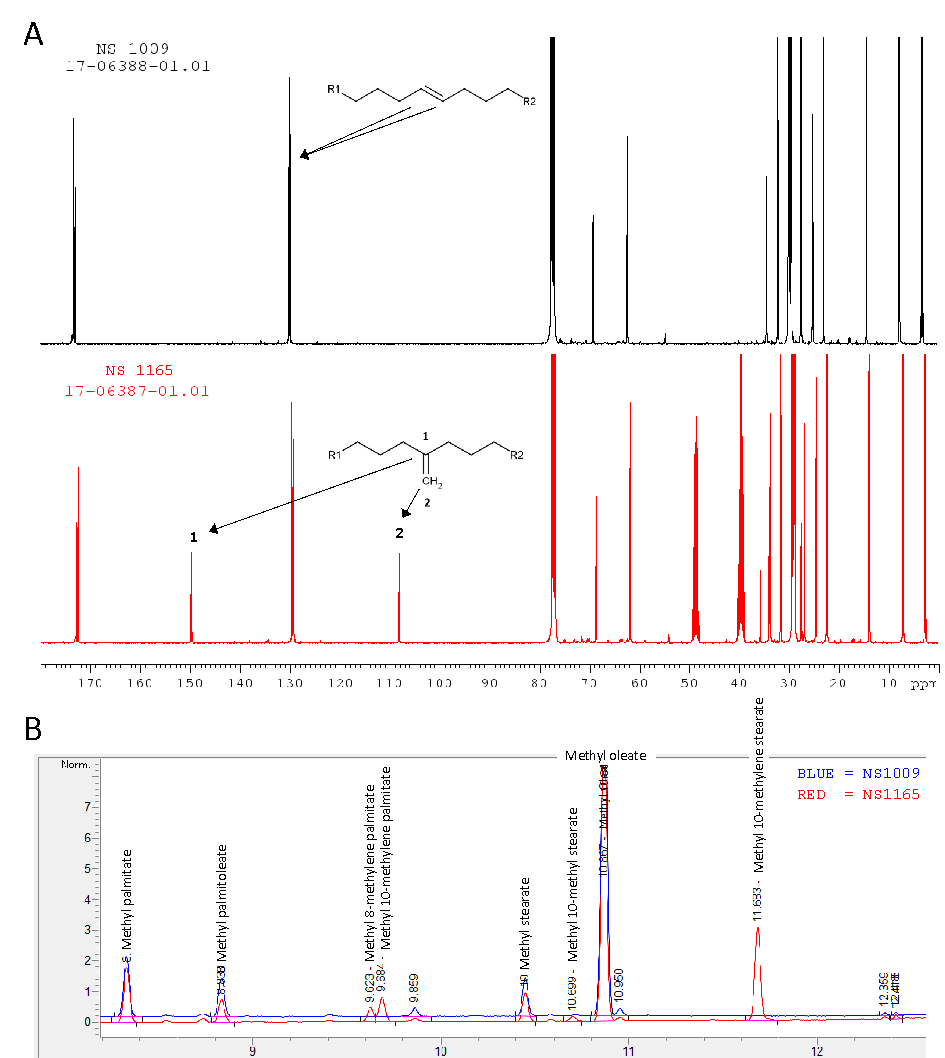


A. Nuclear Magnetic Resonance (NMR) spectra of lipid extracted from NS1165 expressing T. curvata *bfaB* and the parental strain NS1009 (parental control strain) with characteristic ^13^C chemical shifts at 150 and 108 ppm associated with the tertiary and exomethylene alkene carbons. B. Gas chromatography-flame ionization detection (GC-FID) chromatogram of *Y. lipolytica* strains NS1165 and NS1009.

**Figure S2**. *tmpB* gene expression in *S. cerevisiae*

The indicated genes were expressed from 2micron plasmids in *S. cerevisiae* wild-type strain NS20. Six individual transformation colonies for each plasmid transformation were incubated in YPD at 30C for 48 hours. Only 10-methylene palmitate was observed. No 10-methylene stearic acid was detected.

**Figure S3**. Expression of *T. curvata bfaA-B* and *bfaB-A* fusion proteins in *S. cerevisiae*

The *T. curvata bfaA-B and bfaB-A* fusion 2 micron plasmid constructs were expressed in *S. cerevisiae* strain NS20 as in Additional Figure 2. No 10-methylene fatty acids were detected in this experiment.

Figure S4. BfaA-B localization in *S. cerevisiae*.


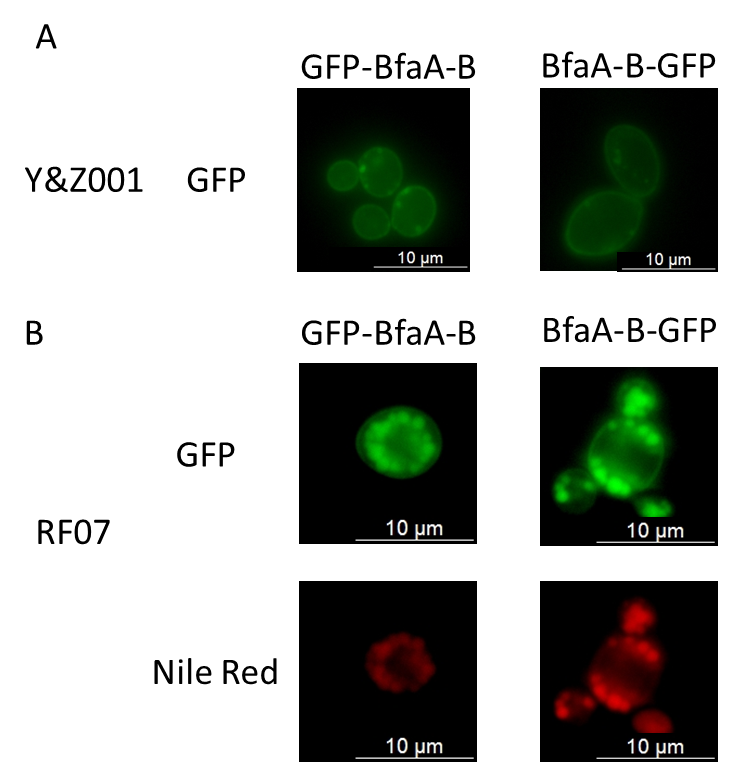


GFP localization of bfaA-B with either N- or C-terminal GFP fusion in *S. cerevisiae* strains. Cells were cultivated for 48 hours in Delft media, diluted and photographed with the fluorescent microscope. A. Strain Y&Z001, a free fatty acid secretion strain. B. Strain RF07 engineered for increased lipid droplet formation. Top two panels are GFP and the bottom two are Nile Red neutral lipid stain.

Figure S5. Production of BFAs in bioreactors.


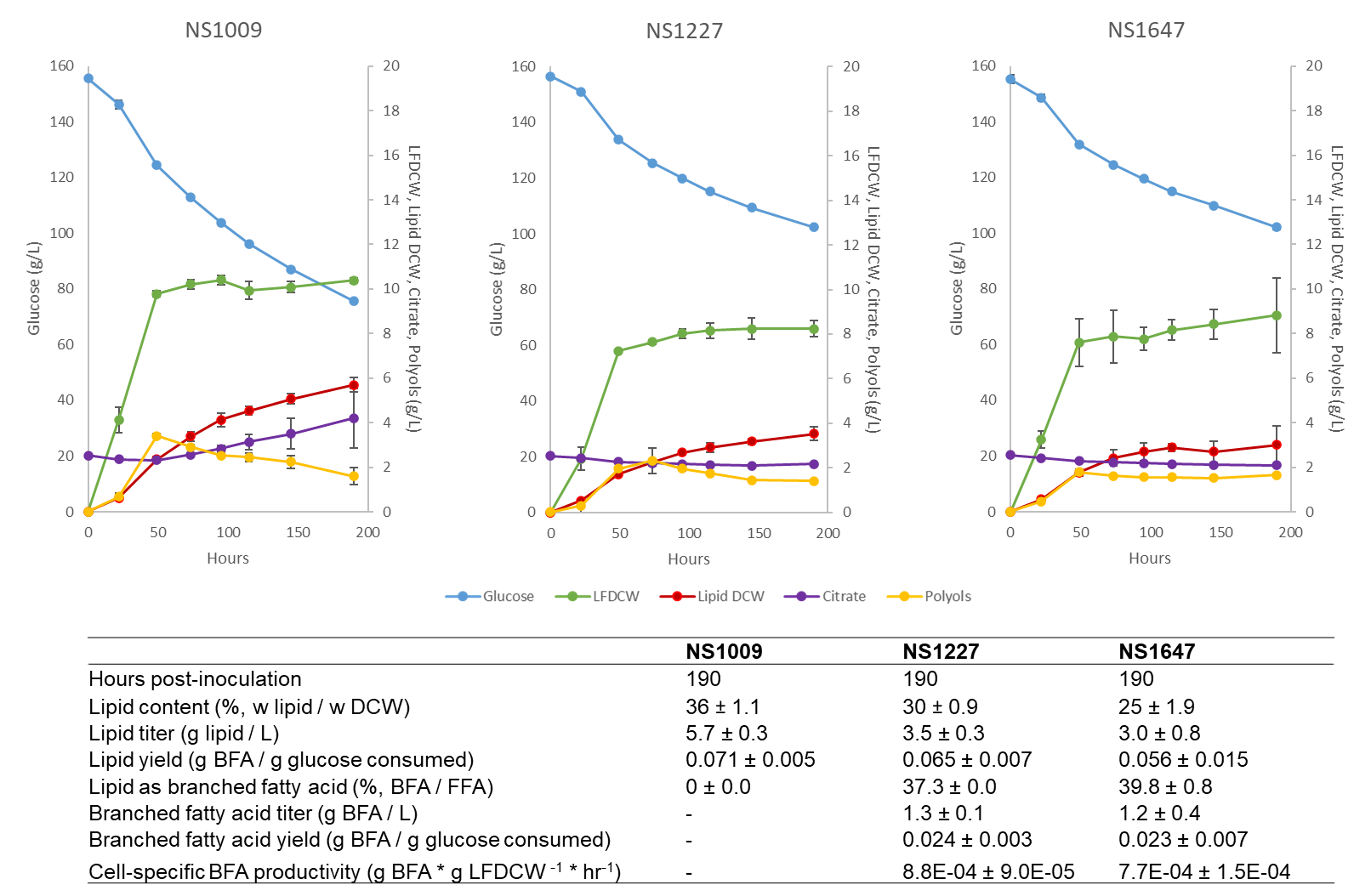


Process parameters were measured for the indicated strains. Glucose consumption, and production of citrate and polyols (erythritol, mannitol and arabitol) were measured by HPLC. Cell growth and lipid accumulation are indicated. Error bars represent the range of data measured for two independent experiments. Summary of the final measurements are presented as a table.
