# Supplementary material for: Production of 10-methyl branched fatty acids in yeast
Source: Biotechnol Biofuels. 2021 Jan 7;14:12. doi: 10.1186/s13068-020-01863-0 (PMC7791843; doi:10.1186/s13068-020-01863-0)
Supplement: Supplementary file 3 — Additional file 3. Sequences and alignments. [file 13068_2020_1863_MOESM3_ESM.docx]

Amino acid sequences used and identified in Tables S1 & S2. Conserved amino acid residues that bind the *E. coli* cyclopropane-fatty-acyl-phospholipid synthase bicarbonate ion are highlighted in both multiple alignments.

>E._coli_cfa

MSSSCIEEVSVPDDNWYRIANELLSRAGIAINGSAPADIRVKNPDFFKRVLQEGSLGLGESYMDGWWECDRLDMFFSKVLRAGLENQLPHHFKDTLRIAGARLFNLQSKKRAWIVGKEHYDLGNDLFSRMLDPFMQYSCAYWKDADNLESAQQAKLKMICEKLQLKPGMRVLDIGCGWGGLAHYMASNYDVSVVGVTISAEQQKMAQERCEGLDVTILLQDYRDLNDQFDRIVSVGMFEHVGPKNYDTYFAVVDRNLKPEGIFLLHTIGSKKTDLNVDPWINKYIFPNGCLPSVRQIAQSSEPHFVMEDWHNFGADYDTTLMAWYERFLAAWPEIADNYSERFKRMFTYYLNACAGAFRARDIQLWQVVFSRGVENGLRVAR

>M_smegmatis_bfaB_MSMEI_RS30765

MTTFKERETSTADRKLTLAEILEIFAAGKEPLKFTAYDGSSAGPEDATMGLDLKTPRGTTYLATAPGDLGLARAYVSGDLEPHGVHPGDPYPLLRALAERMEFKRPPARVLANIVRSIGIEHLKPIAPPPQEALPRWRRIMEGLRHSKTRDAEAIHHHYDVSNTFYEWVLGPSMTYTCACYPTEDATLEEAQDNKYRLVFEKLRLKPGDRLLDVGCGWGGMVRYAARHGVKALGVTLSREQATWAQKAIAQEGLTDLAEVRHGDYRDVIESGFDAVSSIGLTEHIGVHNYPAYFNFLKSKLRTGGLLLNHCITRPDNRSAPSAGGFIDRYVFPDGELTGSGRIITEAQDVGLEVIHEENLRNHYAMTLRDWCRNLVEHWDEAVEEVGLPTAKVWGLYMAGSRLGFETNVVQLHQVLAVKLDDQGKDGGLPLRPWWSA

>M_smegmatis_bfaA_MSMEI_RS30760

VSVVTTDAQAAHAAGVSRLLASYRAIPPSATVRLAKPTSNLFRARARTNVKGLDVSGLTGVIGVDPDARTADVAGMCTYEDLVAATLPYGLAPLVVPQLKTITLGGAVTGLGIESTSFRNGLPHESVLEMDILTGSGEIVTASPDQHSDLFHAFPNSYGTLGYSTRLRIELEPVHPFVALRHLRFHSITDLVAAMDRIIETGGLDGEPVDYLDGVVFSATESYLCVGFKTKTPGPVSDYTGQQIFYRSIQHDGDTGAEKHDRLTIHDYLWRWDTDWFWCSRAFGAQHPVIRRFWPRRLRRSSFYWKLVAYDQRYDIADRIEKRNGRPPRERVVQDVEVPIERCADFVEWFLQNVPIEPIWLCPLRLRDSADGGASWPLYPLKAHHTYVNIGFWSSVPVGPEEGHTNRLIEKKVAELDGHKSLYSDAYYTRDEFDELYGGEVYNTVKKTYDPDSRLLDLYSKAVQRQ

>C_glyciniphilium_bfaB_CGLY_02225

MSRGFTPLTVGQIVDKVITPPAPFRVTAFDGSTAGPADAELALEITSPDALAYIVTAPGDLGLARAYITGSLRVTGDEPGHPYLVFDHLQHLYDQIRRPSAKDLLDIARSLKAMGAIKVQPAPEQETLPGWKRAILEGLSRHSPERDKEVVSRHYDVGNDFYELFLGDSMAYTCAYYPEFDGENQVTGPTGGWRYDDWEKGPTANGPLTQAQDNKHRLVFDKLRLNPGDRLLDVGCGWGGMVRYAARHGVKAIGVTLSREQYEWGKAKIEEEGLQDLAEVRCMDYRDVPESDFDAVSAIGILEHIGVPNYEDYFTRLFAKLRPGGRMLNHCITRPHNRKTKTGQFIDRYIFPDGELTGSGRIITIMQDTGFDVVHEENLRPHYQRTLHDWCELLATNWDQAVHLVGEETARLFGLYMAGSEWGFEHNVIQLHQVLGVKPDAAGSSGVPVRQWWRS

>C_glyciniphilium_bfaA_CGLY_02220

VTVAGRITDAVRIGNGLDQRDLAPVGWYAHEQAVARLKASFDAVPAGRRVRLAKKTSNLFRGRSGEAVGLDVSGLHGVIAVDPVEGTADVQGMCTYEDLVDVLLPYGLAPTVVPQLKTITLGGAVTGMGVESTSFRNGLPHEAVLEMDVLTGTGDILTCSPTQNTDLYRGFPNSYGSLGYSVRLKVRCERVEPYVDLRHVRFDDVQSLTDALDNIVVDKEYEGERVDYLDGVVFSLEESYLVLGRATSEAGPVSDYTRERSYYRSLQHPSGVLRDKLTIRDYLWRWDVDWFWCNRAFGTQNPTIRTLWPRDLLRSSFYWKIIGWDRRFDIADRIEAHNGRPARERVVQDIEVTPDNLPEFLTWFFTHCEIEPVWLCPIRLADDSGERTPWPLYPLSPGDTWVNVGFWSSVPADLMGKDAPTGAFNREVERVVSDLGGHKSLYSEAFYSEEQFAALYGGERPAQLKAVFDPDDRFPGLYEKTVGGV

>C_glyciniphilium_bfaC_CGLY_02230

VAVLCTPLLLGACTIGDAGPGDETTDPVVDTEAPPDKPVPDSAAESGAEDGPDSEVPDDPDQPDAEPVETDPDAPGARGLAIGDCVADMDQLDGTGDIDVVDCAGPHAGEVYAQADIAGKNLFPGNEPLGQEAGAICGGDSFTGYVGIGFPESSLDVVTMMPSKESWAQEDRTVTCVVTDPNLEQIAGTLEQSWR

>M_tuberculosis_bfaB_Rv3720

MAEILEIFTATGQHPLKFTAYDGSTAGQDDATLGLDLRTPRGATYLATAPGELGLARAYVSGDLQAHGVHPGDPYELLKTLTERVDFKRPSARVLANVVRSIGVEHILPIAPPPQEARPRWRRMANGLLHSKTRDAEAIHHHYDVSNNFYEWVLGPSMTYTCAVFPNAEASLEQAQENKYRLIFEKLRLEPGDRLLDVGCGWGGMVRYAARRGVRVIGATLSAEQAKWGQKAVEDEGLSDLAQVRHSDYRDVAETGFDAVSSIGLTEHIGVKNYPFYFGFLKSKLRTGGLLLNHCITRHDNRSTSFAGGFTDRYVFPDGELTGSGRITTEIQQVGLEVLHEENFRHHYAMTLRDWCGNLVEHWDDAVAEVGLPTAKVWGLYMAASRVAFERNNLQLHHVLATKVDPRGDDSLPLRPWWQP

>M_tuberculosis_bfaA_Rv3719

MQGQLSRTRVYTVPVPGSAQSAYACGVERLLASYRSIPATASIRLAKPTSNLFRARVKHDARGLDASGLTGVIGIDPEARTADVAGMCTYEDLIAATLHYGLSPLVVPQLRTITLGGAVTGLGIESASFRNGLPHESVLEMDILTGAGELLTVSPGQHSDLYRAFPNSYGTLGYSTRLRIQLEPVRPFVALRHIRFSSLTAMVAAMERIIDTGGLDGESVDYLDGVVFSADESYLCIGMQTSVPGPVSDYTGQDIYYRSIQHEAGIKEDRLTIHDYFWRWDTDWFWCSRSFGAQNPRLRRWWPRRYRRSSVYWRLMALDQRFGIADRFENSRGRPARERVVQDIEVPIERTCEFLEWFGENVPISPIWLCPLRLRDHAGWPLYPIRPDRSYVNIGFWSSVPVGATEGATNRKIENKVSALDGHKSLYSDSFYTREEFDELYGGETYNTVKKAYDPDSRLLDLYAKAVQRR

>M_phlei_bfaB_MPHLEI_RS13770

MTAIKENPVLTSARKLSLAEILEILAGGELPVRFTAYDGSSAGPADSPLGLELLTPRGTTYLATAPGDLGLARAYIAGDLQPHGVHPGDPYELLKALSEKMEFKRPPAKVLANIVRSIGIEHLKPIAPPPQEAQPRWRRIAEGLRHSKTRDAEAIHHHYDVSNTFYEWVLGPSMTYTCACYPDVDATLEQAQENKYRLVFEKLRLKPGDRLLDVGCGWGGMVRYAAQHGVKAIGVTLSREQATWAQKAIAEQGLSDLAEVRHGDYRDIRESGFDAVSSIGLTEHIGVANYPSYFRFLQSKLRVGGLLLNHCITRPDNKSQASAGGFIDRYVFPDGELTGSGRIIAAAQDVGLEVVHEENLRQHYAMTLRDWCRNLVEHWDEAVAEVGLERAKIWGLYMAGSRLGFETNIVQLHQVLAVKLDRRGGDGGLPLRPWWTP

>M_phlei_bfaA_MPHLEI_RS13765

VSEPRTDARVVQAAGVHKLLESYRAIPPEATVRLAKPTSNLFRARAKTSVKGLDVSGLTHVISVDPDERTAEVAGMCTYEDLVAATLPYGLSPLVVPQLKTITLGGAVTGLGIESASFRNGLPHESVLEMDILTGSGEILTASRDQHPDLFRAFPNSYGTLGYSVRLKIELETVKPFVAVRHLRFHDIEDLVAEMDRIVETGGYDGTPVDYLDGVVFSARESYLTLGFQTATPGPVSDYTGQQIYYRSIQHEDGVKDDRLTIHDYFWRWDTDWFWCSRAFGVQNPTIRRFWPRRLKRSSFYWKLVAYDRKFNIADRIEMHNGRPPRERVVQDIEVPIERVAEFLGWFLDNVPIEPIWLCPLRLRDDAGWPLYPIRAQHTYVNVGFWSSVPVGPTEGHTNRLIERKVSELDGHKSLYSDAYYSRDEFDQLYGGEIYKTVKKAYDPDSRLLDLYAKAVQRQ

>T_curvata_bfaB_TCUR_RS07780

MTLAKVFEELVGADAPVELTAYDGSRAGRLGSDLRVHVKSPYAVSYLVHSPSALGLARAYVAGHLDAYGDMYTLLREMTQLTEALTPKARLRLLAGVLQDPLLRAAASRRLPPPPQEVRTGRTSWFRHTKRRDAKAISHHYDVSNTFYEWVLGPSMTYTCACFPTEDATLEEAQFHKHDLVAKKLGLRPGMRLLDVGCGWGGMVMHAAKHYGVRALGVTLSKQQAEWAQKAIAEAGLSDLAEVRHQDYRDVTEGDFDAISSIGLTEHIGKANLPSYFGFLYGKLKPGGRLLNHCITRPDNTQPAMKKDGFINRYVFPDGELEGPGYLQTQMNDAGFEIRHQENLREHYARTLAGWCRNLDEHWDEAVAEVGEGTARVWRLYMAGSRLGFELNWIQLHQILGVKLGERGESRMPLRPDWGV

>T_curvata_bfaA_TCUR_RS07785

MSQLAVTDHHERAVEALRRSYAAIPPGTPVRLAKQTSNLFRFREPTAAPGLDVSGFNRVLAVDPDARTADVQGMTTYEDLVDATLPHGLMPLVVPQLKTITLGGAVTGLGIESTSFRNGLPHESVLEMQIITGAGEVVTATPDGEHSDLFWGFPNSYGTLGYALKLKIELEPVKPYVRLRHLRFDDAGECAAKLAELSESREHEGDEVHFLDGTFFGPREMYLTLGTFTDTAPYVSDYTGQHIYYRSIQQRSIDFLTIRDYLWRWDTDWFWCSRALGVQNPLIRRVWPKSAKRSDVYRKLVAYEKRYQFKARIDRWTGKPPREDVIQDIEVPAERLPEFLEFFHDKIGMSPVWLCPLRARHRWPLYPLKPGVTYVNAGFWGTVPLQPGQMPEYHNRLIERKVAQLDGHKSLYSTAFYSREEFWRHYDGETYRRLKDTYDPDARLLDLYDKCVRGR

>R_opacus_bfaB_PD630_RS02885

MTTLKASRSQDHKLTIAEILETLSDGMLPLRFSAYDGSAAGPEDAPYGLHLKTTRGTTYLATAPGDLGMARAYVSGDLEARGVHPGDPYEILRVMGDELHFRRPSALTLAAITRSLGWDLLRPIAPPPQEHLPRWRRVAEGLRHSKSRDAEVIHHHYDVSNTFYEYVLGPSMTYTCACYENAEQTLEEAQDNKYRLVFEKLGLQPGDRLLDIGCGWGSMVRYAARRGVKVIGATLSREQAEWAQKAIAEEGLSDLAEVRFSDYRDVPETGFDAISSIGLTEHIGVGNYPAYFGLLQSKLREGGRLLNHCITRPDNQSQARAGGFIDRYVFPDGELTGSGRIITEIQNVGLEVRHEENLREHYALTLAGWCQNLVDNWDACVAEVGEGTARVWGLYMAGSRLGFERNVVQLHQVLAVKLGPKGEAHVPLRPWWK

>R_opacus_bfaA_PD630_RS02880

MREGGRPFRAHRTLPVTGIDAHRAGVERLLASYRAIPTDATVRLAKKTSNLFRARAQTSAPGLDVSGLGGVISVDEQDRTADVAGMCTYEDLVDATLPYGLAPLVVPQLKTITLGGAVTGLGIESTSFRNGLPHESVLEIDVLTGSGDIVTARPEGENSDLFWGFPNSYGTLGYSTRLRIQLEPVKRYVALRHLRFDSLDELQSAMDRIVTERVHDGIPVDYLDGVVFTASESYLTLGHQTDEGGPVSDYTGQNIFYRSIQHSSVNHPKTDKLTIRDYLWRWDTDWFWCSRAFGAQNPTIRRLWPKNLLRSSFYWKLIALDHKYDIGDRLEKRKGNPPRERVVQDVEVPIERTADFVRWFLDEIPIEPLWLCPLRLREPAPAGASSQRPWPLYPLEPKRTYVNIGFWSSVPIVPGRPEGAANRLIEDKVSDFDGHKSLYSDSYYSREDFERLYYGGDRYTELKKRYDPKSRLLDLFSKAVQRR

CLUSTAL multiple sequence alignment by MUSCLE (3.8)

E._coli_cfa ----------------MSSSCIEEVSVPDDNWYRIANELLSRAGIAINGSAPADIRVKNP

C_glyciniphilium_bfaB_CGLY_02225 --------MSRGFTPLTVGQIVDKV-ITPPAPFRVTAFDGSTAG---PADAELALEITSP

T_curvata_bfaB_TCUR_RS07780 ---------------MTLAKVFEEL-VGADAPVELTAYDGSRAG---RLGSDLRVHVKSP

R_opacus_bfaB_PD630_RS02885 MTTLKASR--SQDHKLTIAEILETL-SDGMLPLRFSAYDGSAAG---PEDAPYGLHLKTT

M_tuberculosis_bfaB_Rv3720 -----------------MAEILEIFTATGQHPLKFTAYDGSTAG---QDDATLGLDLRTP

M_smegmatis_bfaB_MSMEI_RS30765 MTTFKERETSTADRKLTLAEILEIF-AAGKEPLKFTAYDGSSAG---PEDATMGLDLKTP

M_phlei_bfaB_MPHLEI_RS13770 MTAIKENPVLTSARKLSLAEILEIL-AGGELPVRFTAYDGSSAG---PADSPLGLELLTP

.. .: . .: * ** .: : : ..

E._coli_cfa DFFKRVLQE-GSLGLGESYMDGWWEC----------------DRLD---------MFFSK

C_glyciniphilium_bfaB_CGLY_02225 DALAYIVTAPGDLGLARAYITGSLRVTGDEPGHPYLVFDHLQHLYDQIRRPSA--KDLLD

T_curvata_bfaB_TCUR_RS07780 YAVSYLVHSPSALGLARAYVAGHLDAY----GDMYTLLREMTQLTE-ALTPKARLRLLAG

R_opacus_bfaB_PD630_RS02885 RGTTYLATAPGDLGMARAYVSGDLEARGVHPGDPYEILRVMGDELH-FRRPSA--LTLAA

M_tuberculosis_bfaB_Rv3720 RGATYLATAPGELGLARAYVSGDLQAHGVHPGDPYELLKTLTERVD-FKRPSA--RVLAN

M_smegmatis_bfaB_MSMEI_RS30765 RGTTYLATAPGDLGLARAYVSGDLEPHGVHPGDPYPLLRALAERME-FKRPPA--RVLAN

M_phlei_bfaB_MPHLEI_RS13770 RGTTYLATAPGDLGLARAYIAGDLQPHGVHPGDPYELLKALSEKME-FKRPPA--KVLAN

: . **:. :*: * :

E._coli_cfa VLRA---GLENQLPHH--------FKDTLRIAGARLFNLQSKKRAWIVGKEHYDLGNDLF

C_glyciniphilium_bfaB_CGLY_02225 IARS--LKAMGAIKVQPAPEQ--ETLPGWKRAILEGLSRHSPERDKEVVSRHYDVGNDFY

T_curvata_bfaB_TCUR_RS07780 VLQDPLLRAAASRRLPPPPQEVRTGRTSW-------F-RHTKRRDAKAISHHYDVSNTFY

R_opacus_bfaB_PD630_RS02885 ITRS--LGWDLLRPIAPPPQE---HLPRWRR-VAEGL-RHSKSRDAEVIHHHYDVSNTFY

M_tuberculosis_bfaB_Rv3720 VVRS--IGVEHILPIAPPPQE---ARPRWRR-MANGL-LHSKTRDAEAIHHHYDVSNNFY

M_smegmatis_bfaB_MSMEI_RS30765 IVRS--IGIEHLKPIAPPPQE---ALPRWRR-IMEGL-RHSKTRDAEAIHHHYDVSNTFY

M_phlei_bfaB_MPHLEI_RS13770 IVRS--IGIEHLKPIAPPPQE---AQPRWRR-IAEGL-RHSKTRDAEAIHHHYDVSNTFY

: . : :: * . ***:.* ::

E._coli_cfa SRMLDPFMQYSCAYW------------------------KDAD-NLESAQQAKLKMICEK

C_glyciniphilium_bfaB_CGLY_02225 ELFLGDSMAYTCAYYPEFDGENQVTGPTGGWRYDDWEKGPTANGPLTQAQDNKHRLVFDK

T_curvata_bfaB_TCUR_RS07780 EWVLGPSMTYTCACF------------------------PTEDATLEEAQFHKHDLVAKK

R_opacus_bfaB_PD630_RS02885 EYVLGPSMTYTCACY------------------------ENAEQTLEEAQDNKYRLVFEK

M_tuberculosis_bfaB_Rv3720 EWVLGPSMTYTCAVF------------------------PNAEASLEQAQENKYRLIFEK

M_smegmatis_bfaB_MSMEI_RS30765 EWVLGPSMTYTCACY------------------------PTEDATLEEAQDNKYRLVFEK

M_phlei_bfaB_MPHLEI_RS13770 EWVLGPSMTYTCACY------------------------PDVDATLEQAQENKYRLVFEK

. .*. * *:** : : * .** * :: .*

E._coli_cfa LQLKPGMRVLDIGCGWGGLAHYMASNYDVSVVGVTISAEQQKMAQERCE--GLD--VTIL

C_glyciniphilium_bfaB_CGLY_02225 LRLNPGDRLLDVGCGWGGMVRYAARH-GVKAIGVTLSREQYEWGKAKIEEEGLQDLAEVR

T_curvata_bfaB_TCUR_RS07780 LGLRPGMRLLDVGCGWGGMVMHAAKHYGVRALGVTLSKQQAEWAQKAIAEAGLSDLAEVR

R_opacus_bfaB_PD630_RS02885 LGLQPGDRLLDIGCGWGSMVRYAARR-GVKVIGATLSREQAEWAQKAIAEEGLSDLAEVR

M_tuberculosis_bfaB_Rv3720 LRLEPGDRLLDVGCGWGGMVRYAARR-GVRVIGATLSAEQAKWGQKAVEDEGLSDLAQVR

M_smegmatis_bfaB_MSMEI_RS30765 LRLKPGDRLLDVGCGWGGMVRYAARH-GVKALGVTLSREQATWAQKAIAQEGLTDLAEVR

M_phlei_bfaB_MPHLEI_RS13770 LRLKPGDRLLDVGCGWGGMVRYAAQH-GVKAIGVTLSREQATWAQKAIAEQGLSDLAEVR

* * ** *:**:*****.:. : * . .* .:*.*:* :* .: ** . :

E._coli_cfa LQDYRDLNDQ-FDRIVSVGMFEHVGPKNYDTYFAVVDRNLKPEGIFLLHTIGSK---KTD

C_glyciniphilium_bfaB_CGLY_02225 CMDYRDVPESDFDAVSAIGILEHIGVPNYEDYFTRLFAKLRPGGRMLNHCITRPHN--RK

T_curvata_bfaB_TCUR_RS07780 HQDYRDVTEGDFDAISSIGLTEHIGKANLPSYFGFLYGKLKPGGRLLNHCITRPDNTQPA

R_opacus_bfaB_PD630_RS02885 FSDYRDVPETGFDAISSIGLTEHIGVGNYPAYFGLLQSKLREGGRLLNHCITRPDN-QSQ

M_tuberculosis_bfaB_Rv3720 HSDYRDVAETGFDAVSSIGLTEHIGVKNYPFYFGFLKSKLRTGGLLLNHCITRHDN-RST

M_smegmatis_bfaB_MSMEI_RS30765 HGDYRDVIESGFDAVSSIGLTEHIGVHNYPAYFNFLKSKLRTGGLLLNHCITRPDN-RSA

M_phlei_bfaB_MPHLEI_RS13770 HGDYRDIRESGFDAVSSIGLTEHIGVANYPSYFRFLQSKLRVGGLLLNHCITRPDN-KSQ

****: : ** : ::*: **:* * ** : :*. * :* * *

E._coli_cfa LNVDPWINKYIFPNGCLP-SVRQIAQSSEPHFVMEDWHNFGADYDTTLMAWYERFLAAWP

C_glyciniphilium_bfaB_CGLY_02225 TKTGQFIDRYIFPDGELTGSGRIITIMQDTGFDVVHEENLRPHYQRTLHDWCELLATNWD

T_curvata_bfaB_TCUR_RS07780 MKKDGFINRYVFPDGELEGPGYLQTQMNDAGFEIRHQENLREHYARTLAGWCRNLDEHWD

R_opacus_bfaB_PD630_RS02885 ARAGGFIDRYVFPDGELTGSGRIITEIQNVGLEVRHEENLREHYALTLAGWCQNLVDNWD

M_tuberculosis_bfaB_Rv3720 SFAGGFTDRYVFPDGELTGSGRITTEIQQVGLEVLHEENFRHHYAMTLRDWCGNLVEHWD

M_smegmatis_bfaB_MSMEI_RS30765 PSAGGFIDRYVFPDGELTGSGRIITEAQDVGLEVIHEENLRNHYAMTLRDWCRNLVEHWD

M_phlei_bfaB_MPHLEI_RS13770 ASAGGFIDRYVFPDGELTGSGRIIAAAQDVGLEVVHEENLRQHYAMTLRDWCRNLVEHWD

. : :.*:**:* * . : .: : : *: * ** * : *

E._coli_cfa EIADNYSERFKRMFTYYLNACAGAFRARDIQLWQVVF----SRGVENGLRVAR----

C_glyciniphilium_bfaB_CGLY_02225 QAVHLVGEETARLFGLYMAGSEWGFEHNVIQLHQVLGVKPDAAGSS-GVPVRQWWRS

T_curvata_bfaB_TCUR_RS07780 EAVAEVGEGTARVWRLYMAGSRLGFELNWIQLHQILGVKLGERGES-RMPLRPDWGV

R_opacus_bfaB_PD630_RS02885 ACVAEVGEGTARVWGLYMAGSRLGFERNVVQLHQVLAVKLGPKGEA-HVPLRPWWK-

M_tuberculosis_bfaB_Rv3720 DAVAEVGLPTAKVWGLYMAASRVAFERNNLQLHHVLATKVDPRGDD-SLPLRPWWQP

M_smegmatis_bfaB_MSMEI_RS30765 EAVEEVGLPTAKVWGLYMAGSRLGFETNVVQLHQVLAVKLDDQGKDGGLPLRPWWSA

M_phlei_bfaB_MPHLEI_RS13770 EAVAEVGLERAKIWGLYMAGSRLGFETNIVQLHQVLAVKLDRRGGDGGLPLRPWWTP

. . .:: *: .. .* . :**.::: * : :

**Highlighted** = Cyclopropane-fatty-acyl-phospholipid synthase bicarbonate ion binding amino acid, conserved in both BfaB and *E. coli* Cfa

* = Single, fully conserved residue in all sequences

: = Amino acids with strongly similar properties

. = Amino acids with weakly similar properties

>D_curvatus_tmpB_B147_RS0124955

MRNQNIKKSINKLLKFAGITVNGNNPYDIQIKNDRLYQRVIHEPALGLGEAYMDQWWECRALDQFMAKVLRANLGEVLKKEWQITWNILKAKLFNQQSSRRAFMVGQSHYDVGNELYQGMLDKQMQYTCGYWKDATTLDQAQEAKLALVCRKLKLAPGMKVLELGCGFGGFAHYAATKYGVEVTGYTVSKEQARFGKELCRGLPVDIRLADYRTATGEYDRVVSIGLMEHVGYKNYGTYMKLTNRLLRDDGIALIHTIGSNASCSACNPWTAKYIFPNGMLPSIAQLGKAMENQFVMEDWHNFGEDYDKTLMAWYENFKQVWPNLEDRYSDRFYRMWEYYLLSCAGGFRSRSMQLWQIVMTKQGTSAPCCRLV

>D_curvatus_tmpA_B147_RS0124950

MIEKEIIIVGGGPAGAACAWKLKQRGITPLVLDKYSFPRPKVCAGWVTPAVFRLLEFQGDDYPYTFSQFDRIHFHMFGIKIPVPTRQYAVRRYEFDAWMISRAHVPVKTHCVKNIIRKNGFYIIDDQYRCRYLIGAGGTHCPVYKTFFTQKRSRPSKSLIVAVEKEYPYDIVHKQCHLWFFDHGLPGYAWYLPKGNNWLNIGIGGKFHRLKQRGQNIMDQWRHFTMDLQKKGFIGENPSSPKGHNYYFQHGPKKYQQDNAFIIGDAAGLSTLDMGEGIHGAVLSGIRAADAIVENKPFTLPHLARFSLPKILLPD

>D_postgatei_tmpB_DESPODRAFT_RS04665

MKNQDIKKSIHKLLNFAGITVNGSNPYDIQVKNDRFYQRIIHEPALGLGEAYMDNWWECRALDQFIAKVLCANLGQVLKKEWRITWNLLTAKLFNQQSSKRAFMVGQRHYDIGNDLYQGMLDKQMQYTCGYWKDATTLDQAQEAKLALVCRKLKLEPGMKVLELGCGFGGFAHYAATRYGVEVTGYTVSKEQVKFAEKLCKGLPVDIRLADYRTATGEYDRVLSIGLMEHVGYKNYGTYMKLTNRLLRDDGIALVHTIGRNDSRCACNSWTAKYIFPNGMLPSIAQLGKAMENQFVMEDWHNFGEDYDKTLMAWYENFRQVWPKLKDRYNDRFYRMWEYYLLSCAGGFRSRSMQLWQIVMTKQGTSAPCCRLV

>D_postgatei_tmpA_DESPODRAFT_RS04670

MINKEIIIVGGGPAGAACAWTLKQKGITPLVLDKYSFPRPKVCAGWITPAVFKLLELRGDDYPYTVSQFDRINFHLFGLKIPVPTRQYAVRRYEFDAWLICRAGVPVNTYCVRNIIRKNGFYIIDDQYQCKYLIGAGGTHCPVYKTFFTQTRPRPPKSLIVAVEKEYPYDISHHQCHLWFFDHGLPGYAWYLPKGNNWLNIGIGAKFHTLKQRGQNIMDQWRHFTMDLQKKGFIRENPPIPKGHNYYFQHGPEKYRQDNAFIIGDAAGLSTLDMGEGIHGAVLSGIRVADAIVDNKPFVLPHPPRFSLPKILLPH

>D_toluolica_tmpB_TOL2_C28310

MNNDKVKHTFHGLMDMAGIKVNGPRPYDIQVKNDNLYQRVLSKAALGLGESYMDQWWECKALDRFIDKILRADLVNKIRQDWNTTWEILKARIINLQKPDRAFMVGQKHYDVGNDLYQAMLDKRMQYTCGYWETADTLESAQKAKLELVCRKIGLKPGMKVLELGCGFGGFARYAAQKYDAHVTGFTVSREQAAFAKKQCRGLPVDIRLDDYRNASGLYDRVVSIGMMEHVGYKNYRAYMELTNRLLKDEGIAFVHTIGSNVSRKICNPWTVKYIFPNSSLPSIAFLGKAMEGLFVVEDWHNFGEDYDKTLMAWHENFKKAWPGLKEKYDERFYRMWTYYLLSCAGGFRSRSMQLWQIVMTKPGRTRPDRRIN

>D_toluolica_tmpA_TOL2_C28300

MIDSKIIIVGGGPAGSACAWKLKQAEEQILILDRKPFPRSKLCAGWINPKALNAIDFKKQEYPFLLHPVDRIHFYLFGVHIPVQTRQYAIRRVEFDDWMVKRANVPVHTHTVKKIIKKNGFYIIDNQYRCQYLVGAGGTHCPVFRVFFSKDEKRPMKSMIAAVEQEYVCDYQDSRCHIWFFDKKLPGYSWYLPKGNGWLNIGIGGKFLKMKKQGTTIIDHWRYFTQKLLRLSLINKIPEMPKGHTYYLRHRMNQCQKDNVFVIGDAAGLSTLDMGEGIHAAIAGGILAAKAIVEKKEFRVESLGKFSLPGMIFQNKRSHKEFF

>M_hydrocarbonclasticus_tmpB_MARHY3375

MAQGTAPKTDYSDSNTKAHVLSLPLENSQADREPHSYERWLIAKLMRMAGSPAIRFQLWNGEVIEPEQGLARFTLHLKDHKALYSLVANPNLAFGDLYSAGRLEIDGDLPDLMESLYRSVHAARQKWPKWLDALWKNHNPRATGISEAKENIHHHYDLGNEFYQLWLDNAEMQYTCAYYEHPGNTLEQAQLAKLEHVCRKLRLRPGMTVVEAGCGWGGLARYMARNYGVKVHSYNISREQLAYAQAESERQGLDGLITYVEDDYRNITGQYDAFVSVGMLEHVGKENYRALSELIKRSLKPNGIALLHSIGRNRPMLMNAWIEKRIFPGAYPPSIGEFMEICEHGDFSVLDVENLRLHYAQTLSHWTERFEANAERVTEMYDEHFTRAWRLYLAGSIAAFRAGSLQLFQVVFTHGDNNQLPQSRQDLYAFPATPEGN

>M_hydrocarbonclasticus_tmpA_MARHY3376

MDHYDVIIVGAGPAGSTLARSLEDAGKNVLVIDKASFPRDKTCAGWVTPAVMESLDINPANYANGRTLQPIRRFRIGMMGQPAVENDHHGIVSYGIRRCEFDAFLLERVRSPKQLATPVKSIVRNNGHWVVNNQWQAPLLIGAGGHFCPVARQLGTGPGKHETVVAAKEVEFEMTPEQADACEARGDTPELWFCRDLKGYAWVFRKGNFLNIGLGREDNHRLTDHLEAFVDDMKHDGRIPADLPGRFKGHAYLLYAHADRPLVDDGVLLIGDAAGLAYTQSGEGIRPAIESALMAAEVILNATDFSAAALQRYGERIAERFGNRASEQAAGWELPEWLKQPVASTLMRSHWFTRKVVTEKWFLHQDVPTLKAVG

>T_halophila_tmpB_SAMN05660831_00818

MQGNTPHGRAKGADLALAERILAGMGNPALAVILWDGSRVGPSDTVADVAVADRRALWAIALNADLHFGDLYAAGRVRIDGDLQTFLETGYRAMDGQPTPWPLRFLHRWQNRPRRNSLNGSRENIHHHYDLGNDFYRLWLDQEVMQYTCAYYPSESASLEEAQIAKLHHVCRKLRLKPGDTVVEAGCGWGGLARFMAKHYGVKVRAFNVSQEQLRFAREEAERQGLSDRVEYVEDDYRNIEGTYDVFVSVGMLEHVGTEQYPELGAVIDRVLAPHGRGLIHTIGRNRPQLMNPWIEKRIFPGAYPPTLREMAAIFEPYAFSIQDVENIRLHYARTLQHWLERFEANVETVRQMFDEHFVRTWRLYLAGSIASFTTGELQLFQTVFTRPDYNELPWSRAYLYTAGEEGA

>T_halophila_tmpA_SAMN05660831_00819

MSERSDVLIVGGGPGGSTLGRALARQGLDVTIVDKQTFPRDKVCAGWVTPAVMESLDLDPNEYARDAVLQPIHAFRTGMLGQRTVVSRYPEPASYGIRRYEFDAWLLERAINDGVRTAQGQPLKELRREDGEWVLNDHLRTPLLIGAGGHFCPVARHLGAAKPGSSETAVHAQEIEFEMTPEQAAACPVEADVPELYFLRDLSGYGWIVRKGDWLNIGLGREGGQKLGEQVSAFAEELKAMGRLHIDPPSKFKGHAYLLHGHSPRPPVHDGALLIGDAAGLAYPQSGEGIRPAVESALMAAEVIGAAGGDYSADRLAAFGDRLQRRFGKGSAAGEPGPVKQALARPLMASSWFARHVILDRWFLHRHQAPLAPAA

CLUSTAL multiple sequence alignment by MUSCLE (3.8)

E._coli_cfa ------------------------MSSSCIEEVSVPDDNWYRIANELLSRAGIAI-----

Mhy_tmpB_MARHY3375 MAQGTAPKTDYSDSNTKAHVLSLPLENSQADREPHSYERW--LIAKLMRMAGSPAIRFQL

Tha_tmpB_SAMN05660831_00818 ------------------------MQGNTPHGRAKGADLA--LAERILAGMGNPALAVIL

Dto_tmpB_TOL2_C28310 ------------------------MNNDKVKH----------TFHGLMDMAGIKV-----

Dcu_tmpB_B147_RS0124955 ------------------------MRNQNIKK----------SINKLLKFAGITV-----

Dpo_tmpB_DESPODRAFT_RS04665 ------------------------MKNQDIKK----------SIHKLLNFAGITV-----

: .. :: *

E._coli_cfa ---------NGSAPADIRVKNPDFFKRVLQEGSLGLGESYMDGWWECDR-LDMFFSKVLR

Mhy_tmpB_MARHY3375 WNGEVIEPEQGLARFTLHLKDHKALYSLVANPNLAFGDLYSAGRLEIDGDLPDLMESLYR

Tha_tmpB_SAMN05660831_00818 WDGSRVG--PSDTVADVAVADRRALWAIALNADLHFGDLYAAGRVRIDGDLQTFLETGYR

Dto_tmpB_TOL2_C28310 ---------NGPRPYDIQVKNDNLYQRVLSKAALGLGESYMDQWWECKA-LDRFIDKILR

Dcu_tmpB_B147_RS0124955 ---------NGNNPYDIQIKNDRLYQRVIHEPALGLGEAYMDQWWECRA-LDQFMAKVLR

Dpo_tmpB_DESPODRAFT_RS04665 ---------NGSNPYDIQVKNDRFYQRIIHEPALGLGEAYMDNWWECRA-LDQFIAKVLC

. : : : : : * :*: * . * :: .

E._coli_cfa A--GLENQLPHHFKDTLRIAGARLFNLQSKKRAWIVGKEHYDLGNDLFSRMLDPF-MQYS

Mhy_tmpB_MARHY3375 SVHAARQKWPKWLDALWKNHNPRATGISEAKENI---HHHYDLGNEFYQLWLDNAEMQYT

Tha_tmpB_SAMN05660831_00818 AMDGQPTPWPLRFLHRWQN-RPRRNSLNGSRENI---HHHYDLGNDFYRLWLDQEVMQYT

Dto_tmpB_TOL2_C28310 A--DLVNKIRQDWNTTWEILKARIINLQKPDRAFMVGQKHYDVGNDLYQAMLDKR-MQYT

Dcu_tmpB_B147_RS0124955 A--NLGEVLKKEWQITWNILKAKLFNQQSSRRAFMVGQSHYDVGNELYQGMLDKQ-MQYT

Dpo_tmpB_DESPODRAFT_RS04665 A--NLGQVLKKEWRITWNLLTAKLFNQQSSKRAFMVGQRHYDIGNDLYQGMLDKQ-MQYT

: .. . . : ***:**::: ** ***:

E._coli_cfa CAYWK-DADNLESAQQAKLKMICEKLQLKPGMRVLDIGCGWGGLAHYMASNYDVSVVGVT

Mhy_tmpB_MARHY3375 CAYYEHPGNTLEQAQLAKLEHVCRKLRLRPGMTVVEAGCGWGGLARYMARNYGVKVHSYN

Tha_tmpB_SAMN05660831_00818 CAYYPSESASLEEAQIAKLHHVCRKLRLKPGDTVVEAGCGWGGLARFMAKHYGVKVRAFN

Dto_tmpB_TOL2_C28310 CGYWE-TADTLESAQKAKLELVCRKIGLKPGMKVLELGCGFGGFARYAAQKYDAHVTGFT

Dcu_tmpB_B147_RS0124955 CGYWK-DATTLDQAQEAKLALVCRKLKLAPGMKVLELGCGFGGFAHYAATKYGVEVTGYT

Dpo_tmpB_DESPODRAFT_RS04665 CGYWK-DATTLDQAQEAKLALVCRKLKLEPGMKVLELGCGFGGFAHYAATRYGVEVTGYT

*.*: . .*:.** *** :* *: * ** *:: ***:**:*.: * .*.. * . .

E._coli_cfa ISAEQQKMAQERCE--GLD--VTILLQDYRDLNDQFDRIVSVGMFEHVGPKNYDTYFAVV

Mhy_tmpB_MARHY3375 ISREQLAYAQAESERQGLDGLITYVEDDYRNITGQYDAFVSVGMLEHVGKENYRALSELI

Tha_tmpB_SAMN05660831_00818 VSQEQLRFAREEAERQGLSDRVEYVEDDYRNIEGTYDVFVSVGMLEHVGTEQYPELGAVI

Dto_tmpB_TOL2_C28310 VSREQAAFAKKQCR--GLP--VDIRLDDYRNASGLYDRVVSIGMMEHVGYKNYRAYMELT

Dcu_tmpB_B147_RS0124955 VSKEQARFGKELCR--GLP--VDIRLADYRTATGEYDRVVSIGLMEHVGYKNYGTYMKLT

Dpo_tmpB_DESPODRAFT_RS04665 VSKEQVKFAEKLCK--GLP--VDIRLADYRTATGEYDRVLSIGLMEHVGYKNYGTYMKLT

:* ** . . ** : *** . :* .:*:*::**** ::* :

E._coli_cfa DRNLKPEGIFLLHTIGSKKTDLNVDPWINKYIFPNGCLPSVRQIAQSSEP-HFVMEDWHN

Mhy_tmpB_MARHY3375 KRSLKPNGIALLHSIGRNRPML-MNAWIEKRIFPGAYPPSIGEFMEICEHGDFSVLDVEN

Tha_tmpB_SAMN05660831_00818 DRVLAPHGRGLIHTIGRNRPQL-MNPWIEKRIFPGAYPPTLREMAAIFEPYAFSIQDVEN

Dto_tmpB_TOL2_C28310 NRLLKDEGIAFVHTIGSNVSRKICNPWTVKYIFPNSSLPSIAFLGKAMEG-LFVVEDWHN

Dcu_tmpB_B147_RS0124955 NRLLRDDGIALIHTIGSNASCSACNPWTAKYIFPNGMLPSIAQLGKAMEN-QFVMEDWHN

Dpo_tmpB_DESPODRAFT_RS04665 NRLLRDDGIALVHTIGRNDSRCACNSWTAKYIFPNGMLPSIAQLGKAMEN-QFVMEDWHN

.* * * ::*:** : . :.* * ***.. *:: : * * : * *

E._coli_cfa FGADYDTTLMAWYERFLAAWPEIADNYSERFKRMFTYYLNACAGAFRARDIQLWQVVFSR

Mhy_tmpB_MARHY3375 LRLHYAQTLSHWTERFEANAERVTEMYDEHFTRAWRLYLAGSIAAFRAGSLQLFQVVFTH

Tha_tmpB_SAMN05660831_00818 IRLHYARTLQHWLERFEANVETVRQMFDEHFVRTWRLYLAGSIASFTTGELQLFQTVFTR

Dto_tmpB_TOL2_C28310 FGEDYDKTLMAWHENFKKAWPGLKEKYDERFYRMWTYYLLSCAGGFRSRSMQLWQIVMTK

Dcu_tmpB_B147_RS0124955 FGEDYDKTLMAWYENFKQVWPNLEDRYSDRFYRMWEYYLLSCAGGFRSRSMQLWQIVMTK

Dpo_tmpB_DESPODRAFT_RS04665 FGEDYDKTLMAWYENFRQVWPKLKDRYNDRFYRMWEYYLLSCAGGFRSRSMQLWQIVMTK

: * ** * *.* : : :.:.* * : ** .. ..* : .:**:* *::.

E._coli_cfa GVENGLRVAR-------------

Mhy_tmpB_MARHY3375 GDNNQLPQSRQDLYAFPATPEGN

Tha_tmpB_SAMN05660831_00818 PDYNELPWSR--AYLYTAGEEGA

Dto_tmpB_TOL2_C28310 PGRTR-PDRR--IN---------

Dcu_tmpB_B147_RS0124955 QGTSA-PCCR--LV---------

Dpo_tmpB_DESPODRAFT_RS04665 QGTSA-PCCR--LV---------

. *

**Highlighted** = Cyclopropane-fatty-acyl-phospholipid synthase bicarbonate ion binding amino acid, conserved in both TmpB and *E. coli* Cfa

* = Single, fully conserved residue in all sequences

: = Amino acids with strongly similar properties

. = Amino acids with weakly similar properties

Sequence for *T. curvata tmsAB* codon optimized for *S. cerevisiae*

ATGTCACAATTAGCAGTTACAGACCATCACGAAAGAGCCGTTGAAGCCTTGAGAAGATCCTACGCCGCCATCCCACCTGGTACTCCTGTTAGATTGGCTAAGCAAACATCTAATTTGTTTAGATTCAGAGAACCAACTGCTGCACCAGGTTTGGATGTTTCAGGTTTTAATAGAGTTTTAGCTGTTGATCCAGATGCTAGAACAGCAGATGTTCAGGGTATGACTACATATGAAGATTTGGTTGATGCAACTTTACCACATGGTTTGATGCCATTGGTTGTTCCACAATTGAAGACTATCACATTAGGTGGTGCTGTTACAGGTTTGGGTATCGAATCTACTTCTTTTAGAAACGGTTTGCCACATGAATCTGTTTTGGAAATGCAAATCATCACAGGTGCTGGTGAAGTTGTTACTGCAACACCAGATGGTGAACATTCTGATTTGTTTTGGGGTTTTCCAAATTCATATGGTACTTTGGGTTACGCATTGAAGTTGAAGATCGAATTGGAACCAGTTAAGCCATACGTTAGATTGAGACATTTGAGATTCGATGATGCTGGTGAATGTGCTGCAAAATTGGCAGAATTATCTGAATCAAGAGAACATGAAGGTGACGAAGTTCATTTCTTGGATGGTACTTTCTTTGGTCCAAGAGAAATGTACTTGACTTTAGGTACTTTTACAGATACTGCTCCATATGTTTCTGATTACACAGGTCAACATATCTATTACAGATCTATTCAACAAAGATCAATCGATTTCTTGACAATCAGAGATTATTTGTGGAGATGGGATACTGATTGGTTTTGGTGTTCAAGAGCTTTGGGTGTTCAAAACCCATTGATCAGAAGAGTTTGGCCAAAATCTGCAAAGAGATCAGATGTTTACAGAAAGTTGGTTGCTTACGAAAAGAGATACCAATTCAAAGCAAGAATTGATAGATGGACTGGTAAACCACCAAGAGAAGATGTTATCCAAGATATCGAAGTTCCAGCAGAAAGATTGCCAGAATTCTTGGAATTTTTCCATGATAAGATTGGCATGTCTCCAGTTTGGTTGTGTCCATTAAGAGCTAGACATAGATGGCCATTGTATCCATTAAAACCAGGTGTTACATACGTTAATGCAGGTTTTTGGGGTACTGTTCCATTGCAACCAGGTCAAATGCCAGAATACCATAACAGATTGATCGAAAGAAAGGTTGCTCAATTGGATGGTCATAAGTCTTTGTACTCAACAGCATTCTACTCAAGAGAAGAATTTTGGAGACATTATGATGGTGAAACATACAGAAGATTGAAGGATACTTACGATCCAGATGCTAGATTGTTAGATTTGTACGATAAATGTGTCAGAGGTAGAATGACTTTAGCCAAGGTATTTGAAGAATTGGTCGGTGCTGATGCCCCTGTAGAATTGACTGCTTATGACGGTTCCAGAGCCGGTAGATTGGGTTCTGATTTGAGAGTTCATGTTAAATCTCCATATGCTGTTTCATACTTAGTTCATTCTCCATCAGCATTGGGTTTAGCAAGAGCTTATGTTGCTGGTCATTTGGATGCATATGGTGACATGTACACTTTGTTAAGAGAAATGACTCAATTGACAGAAGCTTTGACACCAAAGGCAAGATTGAGATTGTTAGCTGGTGTTTTGCAAGATCCATTGTTAAGAGCTGCAGCTTCTAGAAGATTGCCACCACCACCACAAGAAGTTAGAACTGGTAGAACATCATGGTTCAGACATACTAAGAGAAGAGATGCAAAGGCTATCTCTCATCATTACGATGTTTCAAACACATTCTACGAATGGGTTTTGGGTCCATCTATGACTTACACATGTGCTTGTTTTCCAACTGAAGATGCTACATTGGAAGAAGCACAATTCCATAAGCATGATTTGGTTGCTAAGAAATTGGGTTTAAGACCAGGCATGAGATTGTTAGATGTTGGTTGTGGTTGGGGTGGTATGGTTATGCATGCAGCTAAACATTATGGTGTTAGAGCTTTGGGTGTTACTTTATCTAAACAACAAGCTGAATGGGCACAAAAAGCAATTGCTGAAGCAGGTTTGTCAGATTTGGCAGAAGTTAGACATCAAGATTACAGAGATGTTACTGAGGGTGACTTCGATGCTATCTCTTCAATCGGTTTAACAGAACATATCGGTAAAGCAAATTTGCCATCATACTTCGGTTTCTTGTACGGTAAATTGAAGCCAGGTGGTAGATTGTTGAACCATTGTATCACTAGACCAGATAACACACAACCAGCTATGAAGAAAGATGGTTTTATTAACAGATACGTTTTCCCAGATGGTGAATTAGAAGGTCCAGGTTACTTGCAAACTCAAATGAATGATGCTGGTTTCGAAATCAGACATCAAGAAAATTTGAGAGAACATTACGCTAGAACATTAGCAGGTTGGTGTAGAAATTTGGATGAACATTGGGATGAAGCTGTTGCAGAAGTTGGTGAAGGTACAGCTAGAGTTTGGAGATTGTACATGGCAGGTTCTAGATTGGGTTTCGAATTGAACTGGATCCAATTGCATCAAATCTTGGGTGTTAAGTTGGGTGAAAGAGGTGAATCAAGAATGCCATTGAGACCTGATTGGGGTGTT
